# Structural and biochemical characterisation of a novel alginate lyase from *Paenibacillus* sp. str. FPU-7

**DOI:** 10.1038/s41598-019-51006-1

**Published:** 2019-10-16

**Authors:** Takafumi Itoh, Emi Nakagawa, Moe Yoda, Akari Nakaichi, Takao Hibi, Hisashi Kimoto

**Affiliations:** Department of Bioscience and Biotechnology, Fukui Prefectural University, 4-1-1 Matsuoka Kenjyoujima, Eiheiji-cho, Yoshida-gun, Fukui 910-1142 Japan

**Keywords:** Polysaccharides, Enzymes, X-ray crystallography, Bacterial structural biology

## Abstract

A novel alginate lyase, PsAly, with a molecular mass of 33 kDa and whose amino acid sequence shares no significant similarity to other known proteins, was biochemically and structurally characterised from *Paenibacillus* sp. str. FPU-7. The maximum PsAly activity was obtained at 65 °C, with an optimum pH of pH 7–7.5. The activity was enhanced by divalent cations, such as Mg^2+^, Mn^2+^, or Co^2+^, and inhibited by a metal chelator, ethylenediaminetetraacetic acid. The reaction products indicated that PsAly is an endolytic enzyme with a preference for polymannuronate. Herein, we report a detailed crystal structure of PsAly at a resolution of 0.89 Å, which possesses a β-helix fold that creates a long cleft. The catalytic site was different from that of other polysaccharide lyases. Site-directed mutational analysis of conserved residues predicted Tyr184 and Lys221 as catalytic residues, abstracting from the C5 proton and providing a proton to the glycoside bond, respectively. One cation was found to bind to the bottom of the cleft and neutralise the carboxy group of the substrate, decreasing the p*K*_a_ of the C5 proton to promote catalysis. Our study provides an insight into the structural basis for the catalysis of alginate lyases and β-helix polysaccharide lyases.

## Introduction

Polysaccharides, such as cellulose, starch, chitin, and alginate, are widely distributed in nature and constitute renewable biopolymer resources with a potential for bioconversion into many useful biological and chemical products. Identifying new enzymes for the synthesis, degradation, and modification of these polysaccharides is an important task for bioconversion. Microbes possess many polysaccharide-degrading enzymes with metabolic pathways that depend on their habitats. Polysaccharide-degrading enzymes, such as glycoside hydrolases and polysaccharide lyases, are classified into families in the CAZy database^1^ based on their primary structures. These degrading enzymes have been isolated from microbial populations based on their enzymatic activities. However, a more powerful tool for the detection of novel enzymes is whole-genome sequencing^2,3^.

As reported previously, we have isolated a Gram-positive bacterium, *Paenibacillus* sp. str. FPU-7 (*P*. str. FPU-7), a chitinolytic bacterium, and characterised the secreted chitinases of the bacterium^4^. The genus *Paenibacillus* is the largest genus of aerobic endospore-forming bacteria (after *Bacillus*), is widely distributed in the environment, including in the air, rhizosphere, soil, and marine environments, and has diverse physiological characteristics, including several polysaccharide-degrading enzymes^5^. The draft genome sequence of *P*. str. FPU-7 suggested that the bacterium potentially contains enzymes with novel functions^4,6,7^. In this study, we experimentally determined the function of a putative protein, as a novel alginate lyase (PsAly), whose gene is found beside two characterised chitinase genes (*chiC* and *chiD*) in the genome.

Alginate, a linear anionic polysaccharide, is widely distributed in the cell walls of brown algae and is synthesised by several bacteria, including *Pseudomonas aeruginosa* and *Azotobacter vinelandii*. The polysaccharide is a heteropolymer consisting of two uronic acids, α-L-guluronic acid (G) and β-D-mannuronic acid (M). These residues are covalently linked with 1,4-glycosidic bonds in three blocks: a homopolymeric G block (PG), a homopolymeric M block (PM), and a heteropolymeric block (MG)^8^. In brown algae and alginate-producing bacteria, the M residue can be epimerised to its C5 epimer, the G residue. In polymer form, alginate is used as a biomaterial for hydrogels in medical applications, including tissue engineering and drug delivery, thanks to its biocompatibility and gelation abilities. Alginate oligosaccharides are composed of 3 to 25 monomers and have several biological properties, including antioxidant, neuroprotective, antibacterial, and antitumor capacities^9^, as well as promoting growth and root elongation in plants^10^. Bacterial alginate lyases can degrade alginate into oligosaccharides or monosaccharides. Lyases catalyse a β-elimination reaction of glycoside bonds to yield a 4,5-unsaturated sugar, 4-deoxy-L-erythro-hex-4-enopyranosyluronic acid, at the non-reducing end^11^. Due to their industrial potential, a number of alginate-degrading bacteria and alginate lyases have been isolated and identified. These alginate lyases are classified into 10 polysaccharide-degrading enzyme families (PLs) in the CAZy database: PL5, PL6, PL7, PL14, PL15, PL17, PL18, PL32, PL34, and PL36^11^. These families have the same lyase activities but diverse structures; PL5, PL15, and PL17 share an (α/α)_6_-barrel fold as a catalytic domain; PL6 has a β-helix fold; PL7, PL14, and PL18 alginate lyases share a β-jelly roll fold. Alginate lyases generally degrade alginates into oligosaccharides in an endolytic manner^12,13^, while some exotype lyases degrade alginates in an exolytic manner^14–16^. Each alginate lyase has a different substrate specificity; some alginate lyases prefer PM, whereas others prefer PG or MG^11^.

In this paper, we describe the functions and structure of a novel alginate lyase, PsAly. The crystal structure of this alginate lyase was determined at a resolution of 0.89 Å. The basic structural fold is a single right-handed β-helix fold. Right-handed β-helix folds are commonly found in carbohydrate-active enzymes. The number of coils (10 coils for PsAly) is different among these β-helix folds, and the active site architecture is different from that of other polysaccharide lyases with β-helix folds. Amino acid sequence similarity and site-directed mutagenesis studies indicated the putative residues involved in substrate binding and catalysis. Our results provide an insight into the structural basis for the catalysis of alginate lyase and β-helix fold polysaccharide lyases.

## Results and Discussion

### *In silico* cloning of a protein with unknown function from *P*. str. FPU-7

*P*. str. FPU-7 is a known chitin-degrading bacterium^4^. In this bacterium, seven chitinase genes (*chiA-F and W*) have been cloned and their enzymatic properties have been characterised^4,6,7,17^. In this study, we re-examined the draft genome of *P*. str. FPU-7 and detected a gene (accession number: LC490364) with 1,014 bp and a GC content of 55.7% for a hypothetical protein of unknown function composed of 337 amino acid (aa) residues next to the chitinase genes *chiC* and *chiD* (Fig. S1). A signal peptide sequence (35 aa residues; Met1 to Ala35) in the form of an extracellular secretion was detected at the N-terminal region of this gene product using the SignalP^18^ program. The other amino acid sequence (302 aa residues; Ala36 to Asn337) showed no significant sequence identity (<30%) with those of any functionally characterised proteins classified using the Pfam^19^ database or BLAST^20^ search program. To identify the function of this protein, the recombinant protein without the signal peptide was prepared using an *E*. *coli* expression system.

### Identification of the gene product to alginate lyase

**Figure 1:**
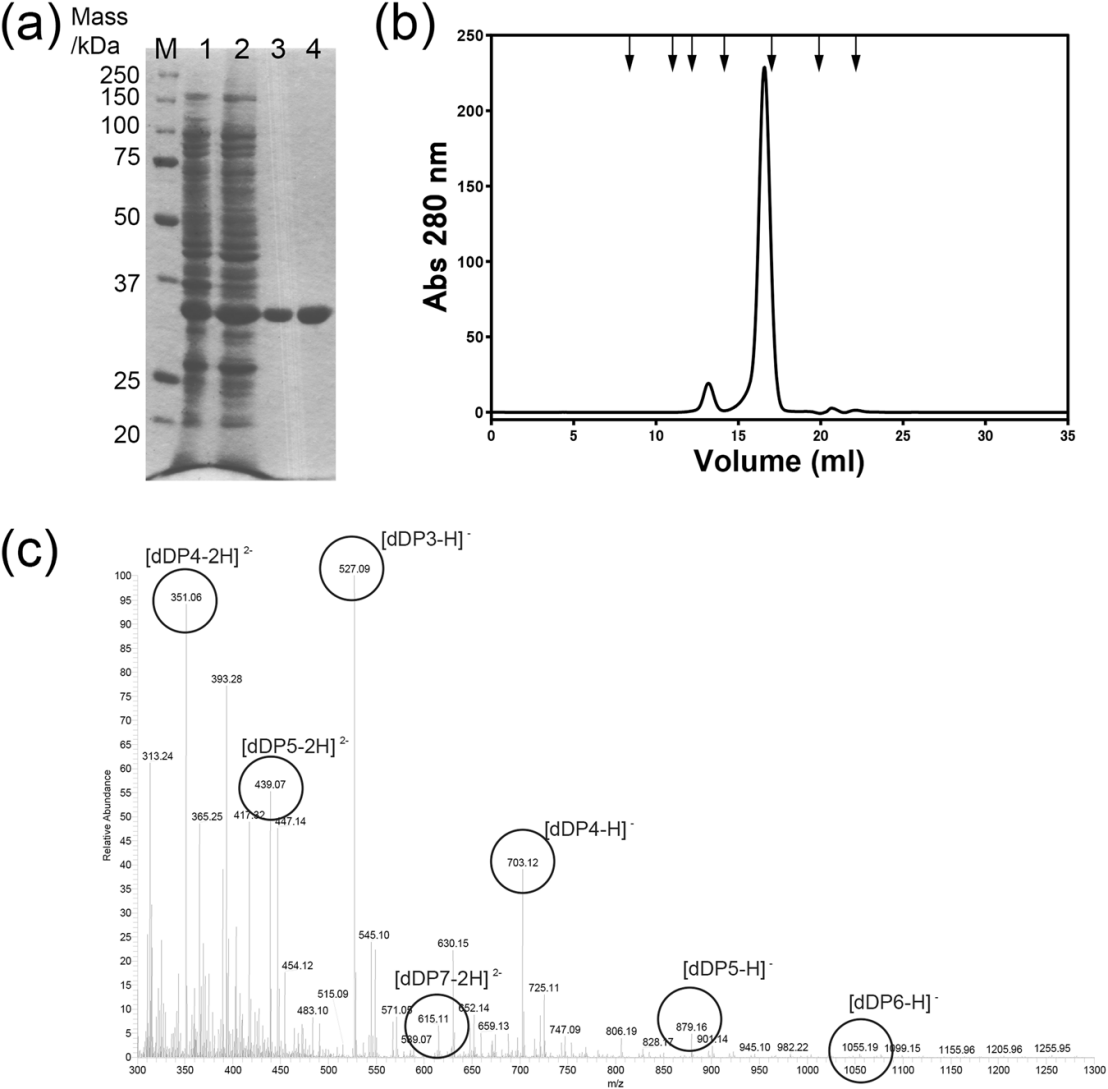
(**a**) SDS-PAGE profile of recombinant PsAly expressed in *E*. *coli* following the purification scheme, (**b**) gel permeation chromatography analysis of PsAly, and (**c**) mass spectrum (negative-ESI MS) of the reaction products with PsAly and alginate. (**a**) Protein bands were stained with CBB R-250. Lane M, molecular mass standards; lane 1, cell extract (50 μg) of *E*. *coli* harbouring the expression plasmid; lane 2, aliquot (5 μg) of elution from Ni-IMAC (HisTrap HP); lane 3, aliquot (5 μg) of elution from AEC (HiTrapQ). (**b**) Arrows indicate the elution positions of molecular mass markers; from left to right: 2,000, 440, 134, 67, 12, 1.355, and 0.376 kDa. (**c**) The resulting peaks in the MS spectrum correspond to unsaturated alginate oligosaccharide (dDP3, dDP4, and dDP5) [M-H]^−^ or [M-2H]^2−^ ions. The small peaks of the ions corresponding to dDP6 and dDP7 were also observed as [M-H]^−^ or [M-2H]^2−^ ions in the spectrum.

A protein with a molecular mass of 33 kDa, based on sodium lauryl sulphate polyacrylamide gel electrophoresis (SDS-PAGE), including a C-terminal His6 tag, was purified by immobilised metal affinity chromatography and anion-exchange chromatography (Fig. 1(a)). Analysis of the N-terminal amino acid residues (ATRTI) by protein sequencing showed that the first Met residue derived from the expression vector was removed. The purified protein was expressed as a monomer, according to gel permeation chromatography (Fig. 1(b)). The gene of this protein is located beside two chitinase genes, *chiC* and *chiD*. As such, the degradation activity of several polysaccharides (chitin, chitosan, curdlan, cellulose, carboxymethyl cellulose, xylan, laminarin, alginate, pectin, sodium heparin, xanthan, and gellan) were examined by detecting the increase of the reducing ends of the reaction products using 3,5-dinitrosalicylic acid (DNS). The recombinant protein exhibited significant degradation activity for alginate (Fig. S2). This protein specifically acted on alginate and not on any other uronic acid-containing polysaccharide, including pectin, heparin, xanthan, and gellan. Almost all of the characterised alginate-degrading bacteria can depolymerise alginate into oligosaccharides using alginate lyases, not hydrolases. Alginate lyases degrade alginate via a β-elimination mechanism, producing unsaturated uronic acid oligosaccharides with double bonds between the C4 and C5 carbons of the sugar rings at the non-reducing terminus with an absorbance maximum at 235 nm^11^. The reaction products of this recombinant protein developed a red colour in the DNS assay (Fig. S2) and exhibited a strong absorbance at 235 nm. These results suggest that the reaction products of this protein have the aldehyde groups at the reducing ends and double bonds at the non-reducing ends. Mass spectrum analysis (negative-ESI MS; Fig. 1(c), Table S1, and MS/MS; Fig. S3) indicated that the final products obtained by this protein were unsaturated oligosaccharide mixtures composed mainly of a trimer to pentamer (dDP3, dDP4, and dDP5). In this study, unsaturated trisaccharide of alginate is referred to as dDP3 (degree of polymerisation). Therefore, we identified this purified protein as an alginate lyase; hereinafter, this protein is denoted as PsAly, and its gene as *aly*.

### Enzymatic characteristics of PsAly

**Figure 2:**
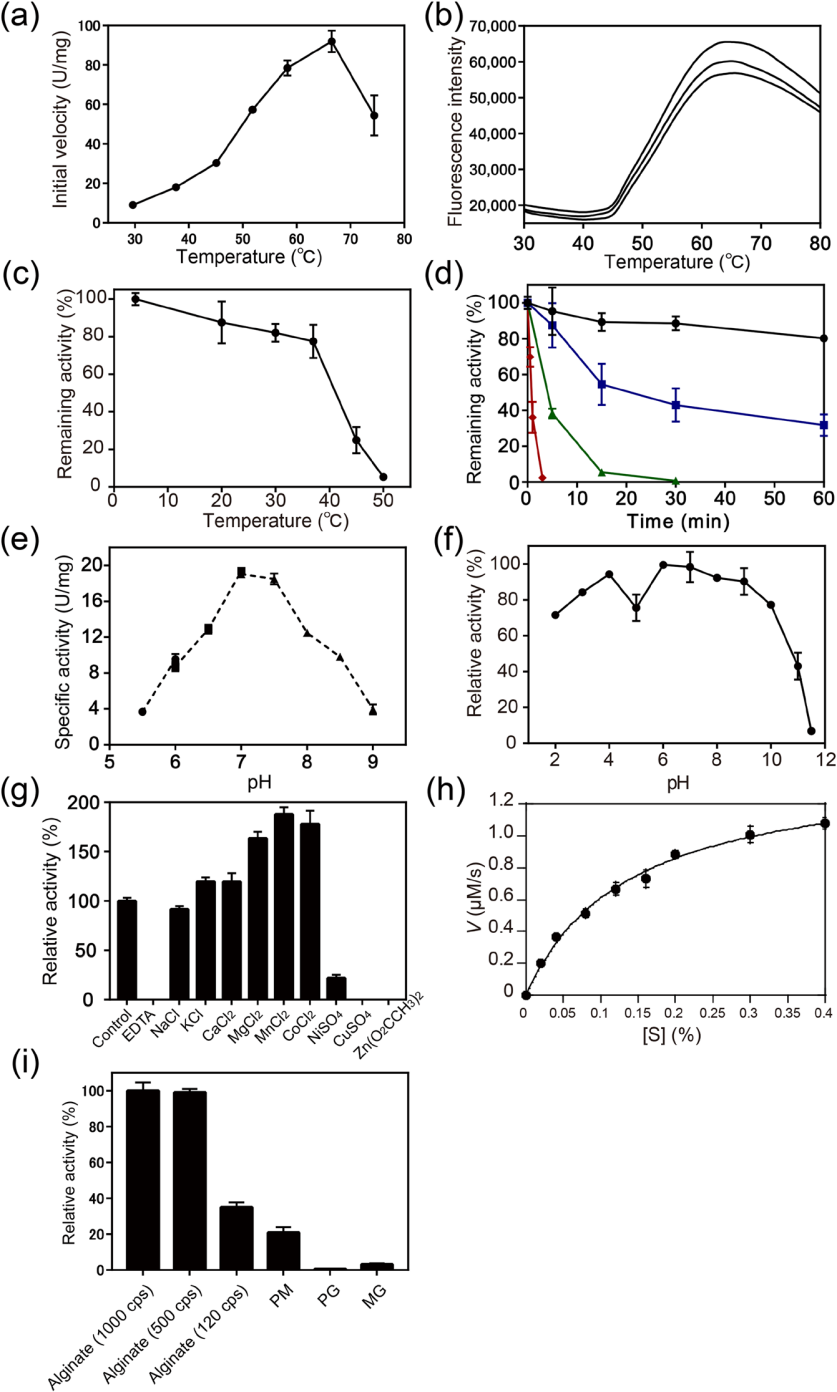
Biochemical properties of PsAly. The vertical error bars on the data points represent the standard deviation of the mean. (**a**) The activity-temperature profile of PsAly was evaluated by the initial velocity (U/mg) at each temperature. (**b**) The thermal stability of PsAly was evaluated by differential scanning fluorimetry by measuring changes in the fluorescence of a dye (SYPRO Orange). The melting temperature (*T*_m_) of PsAly was calculated as an inflection point of the melt curve (*T*_m_ = 52.9 ± 0.4 °C). Assays were performed in triplicate. (**c**) The thermal stability of PsAly was evaluated by the determination of the remaining activities (%) after incubation for 1 h at various temperatures. (**d**) The kinetics of the thermal inactivation of PsAly at 37 °C (black circle), 47 °C (blue square), 57 °C (green triangle), or 67 °C (red diamond) were evaluated by measuring the remaining activity (%) at appropriate intervals. (**e**) The pH-activity profile of PsAly was evaluated by the specific activity (U/mg). (**f**) The pH stability of PsAly was evaluated by the determination of the remaining activities (%) after incubation for 1 h at various pH levels. (**g**) The effect of the cations and metal chelation on enzyme activity was determined by adding the listed reagents. **(h**) The kinetic properties (*k*_cat_ and *K*_m_) were determined by fitting to the Michaelis–Menten equation. (**i**) The substrate specificities were determined using 0.4% (w/v) sodium alginate (1,000, 500, or 120 cps), PM, PG, or MG as the substrate.

**Figure 3:**
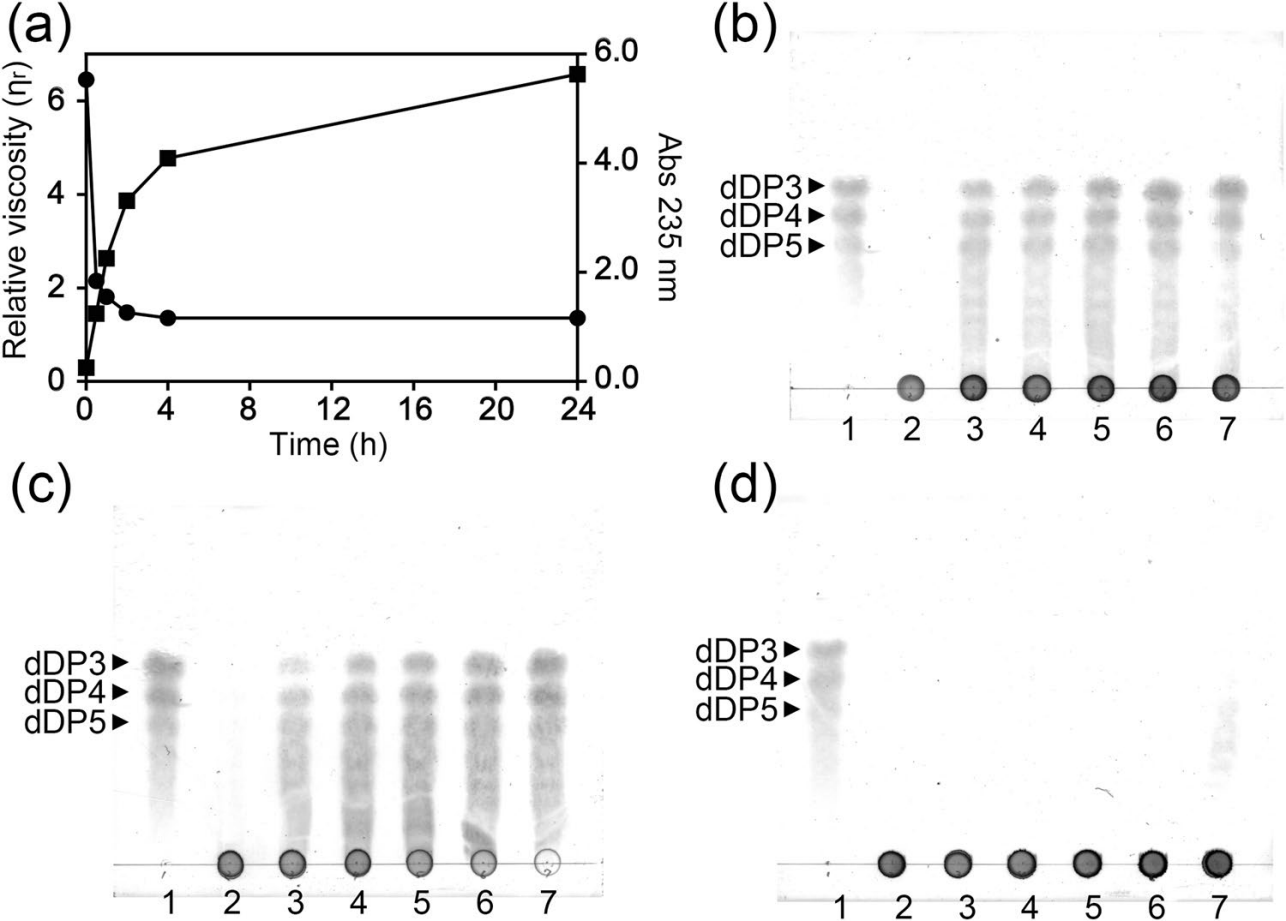
Enzymatic reaction mode of PsAly. (**a**) The enzyme reaction products were analysed by the viscosity (black circle) and absorbance at 235 nm (black box) of the solution with PsAly and alginate after incubation at 37 °C for 0, 0.5, 1, 2, 4, and 24 h. (**b**–**d**) The reaction products with PsAly and alginate (**b**), PM (**c**), or PG (**d**) were also visualised by TLC. Lane 1, 20 μg of oligosaccharide mixtures (dDP3, dDP4 and dDP5); Lanes 2–7, the reaction products (20 μg) at 0, 0.5, 1, 2, 4, and 24 h, respectively.

The apparent highest activity of PsAly was initially obtained at 67 °C from the activity-temperature profile by measuring the initial velocities at various temperatures (Fig. 2(a)), whereas the protein thermal stability of PsAly was evaluated by differential scanning fluorimetry (Fig. 2(b)), which monitored thermally induced protein denaturation by measuring changes in the fluorescence of a dye (SYPRO Orange) that binds to hydrophobic regions of a protein that are exposed by unfolding. The melting temperature (*T*_m_) of PsAly was 52.9 ± 0.4 °C. After preincubation at temperatures from 4 to 50 °C for 1 h, significant decreases in activities were observed over 37 °C, although the enzyme was relatively stable at temperatures from 4 to 37 °C (Fig. 2(c)). The kinetic parameters of the thermal inactivation of PsAly were investigated at 37, 47, 57, and 67 °C (Fig. 2(d)). The activity of PsAly was retained at approximately 80% of the highest activity after incubation for 60 min at 37 °C. After incubation for 24 h at 37 °C, the enzyme was stable at more than 50% of the relative activity (Fig. S4). The half-lives (50% loss of activity) of PsAly at 47, 57, and 67 °C were approximately 15 min, 3 min, and 40 s, respectively. The optimal pH was 7.0 to 7.5 (Fig. 2(e)) and the enzyme retained more than 70% of the highest activity after incubation for 1 h in a buffer with a pH range of 2–10 (Fig. 2(f)). The enzyme activity was significantly increased to 160%–180% by several divalent cations, including 5 mM Mg^2+^, Mn^2+^, or Co^2+^, although the activity was strongly inhibited by 5 mM EDTA, Ni^2+^, Cu^2+^, or Zn^2+^ (Fig. 2(g)). The specific activity for 0.4% (w/v) alginate was 18 ± 1.3 U/mg at 37 °C in 50 mM Tris buffer pH 7.5. The kinetic properties, *k*_cat_ (14.0 ± 0.6 1/s) and *K*_m_ (0.14 ± 0.01% [w/v]), were determined using the Michaelis–Menten equation (Fig. 2(h)). In general, alginate lyases are classified as either mannuronate lyase (EC 4.2.2.3) or guluronate lyase (EC 4.2.2.11), which preferentially act on PM or PG chains, respectively. PsAly preferentially degraded the glycosidic bond of PM over PG and MG (Fig. 2(i)). However, the enzyme showed greater activity on alginate than PM. PM, PG, and MG were prepared from commercially available alginate by hydrolysis using hydrochloric acid in the laboratory. The chain lengths of the hydrolysed products (<30 DP) were shorter than those of the original alginate (approximately 1,000–2,000 DP)^21^. This result indicates that PsAly prefers the PM region of the longer chain alginate. The mode of the enzymatic reaction was determined by measuring the viscosity, the absorbance at 235 nm (Fig. 3(a)), and by thin layer chromatography (TLC) (Fig. 3(b–d)). The viscosity of alginate was found to rapidly decrease, whereas the absorbance at 235 nm increased moderately (Fig. 3(a)). TLC indicated that the oligosaccharides, in addition to the final products, tri-, tetra- and penta-saccharides, were produced during the reaction with alginate (Fig. 3(b)) and PM (Fig. 3(c)). TLC also indicated that PsAly was unable to degrade PG (Fig. 3(d)). These results suggest that PsAly can degrade alginate oligosaccharides longer in chain-length than a hexamer in an endolytic manner. Since alginate is a mixture of heterogeneous polymers with different contents of PG and PM, it is difficult to determine the specific activities or the kinetic parameters (*k*_cat_ and *K*_m_) of alginate lyases using a single species of substrate. However, the specific activity of PsAly is similar to those of other well-studied alginate lyases^14,22–27^ (Table S2). Most alginate lyases exhibit maximal activities at pH 7 to 9 (Table S2). The optimal pH (7.0 to 7.5) of PsAly was also within this range. The *K*_m_ value of PsAly (0.14% or 7.9 mM of monomer uronic acid) is similar to those of other alginate lyases. For example, the *K*_m_ values of FsAlyPL6 of *Flammeovirga* sp. NJ-04^24^, Aly-SJ02 of *Pseudomonas* sp. SM0524^27^, AlgMsp of *Microbulbifer* sp. 6532A^28^, and AlyA1PL7 of *Zobellia galactanivorans*^29^ are 0.050% to 0.16% (varied with substrates), 0.047% to 0.28%, 3.4 mM, and 1.7 to 6.2 mM, respectively. The catalytic rate constant (*k*_cat_ = 14 1/s) of PsAly is also similar to those of other alginate lyases, such as FsAlyPL6^24^ (5.0 to 63 1/s), AlgMsp^28^ (42 1/s), and AlyA1PL7^29^ (13 to 20 1/s).

### Overall crystal structure of PsAly

**Figure 4:**
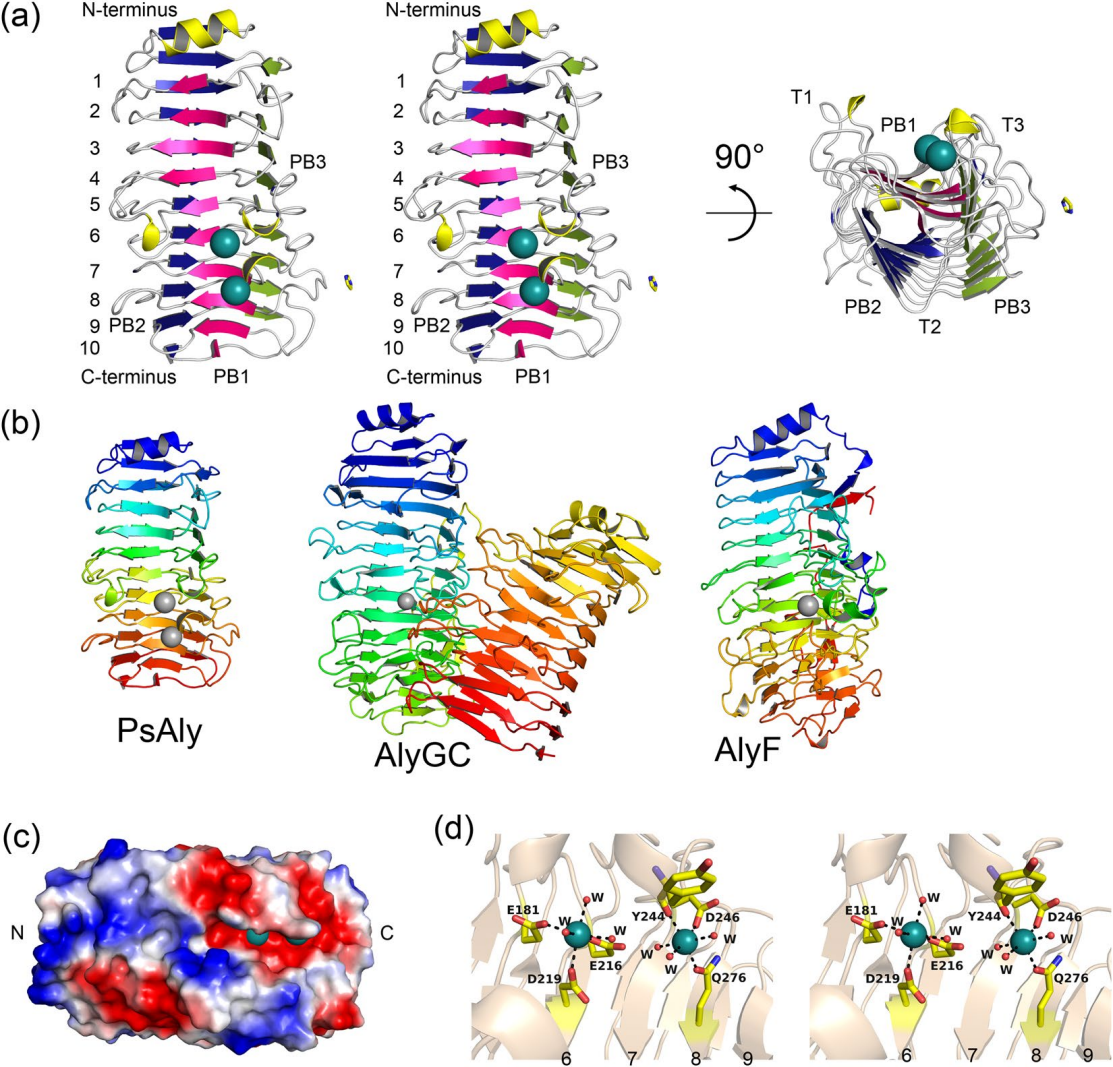
Crystal structure of PsAly. (**a**) The overall structure of PsAly is represented by a ribbon model. The β-helix structure has 10 coils and is formed by three distorted β-sheets, named PB1 (pink), PB2 (blue), and PB3 (yellow). The β-strands in the helix are connected by three turns, T1 (between PB1 and PB2), T2 (between PB2 and PB3), and T3 (between PB3 and PB1). The two sodium ions are represented by deep teal balls and the bound imidazole is represented by a yellow stick model. (**b**) The structural comparisons between PsAly (left) and PL6 alginate lyases (AlyGC [centre] and AlyF [right]). The structures are represented by rainbow ribbon models. The metal ions are represented by grey balls (PsAly and AlyF: sodium ions and AlyGC: calcium ion). (**c**) The electrostatic potentials at pH 7 are also represented. The +55 to −55 kT/e potential isocontours are shown as blue to red surfaces, respectively. (**d**) Close-up view of bound sodium ions and surrounding residues. Two sodium ions (deep teal balls) were located on the cleft and chelated by Glu181, Glu216, Asp219, Tyr244, Asp246, and Gln276 residues and six water molecules (stick and ball models).

To determine the structural characteristics of PsAly, the crystal structure of the enzyme was determined at an ultra-high resolution of 0.89 Å using the single-wavelength anomalous diffraction of selenium (Se-SAD) (Fig. 4(a)). The crystallographic statistics for data collections and structure refinement are summarised in Table 1. The refined model comprised 298 residues (Ala36 to Ala333) for one protein molecule in an asymmetric unit. All amino acid residues were identified in a 2*F*_o_–*F*_c_ map, except for 12 C-terminal residues (Ala334 to Asn337 [APAN] and Leu338 to His345 derived from the plasmid vector [LEHHHHHH]). PsAly folds into a right-handed β-helix structure comprising 10 β-stranded coils and one α-helix cap at the N-terminus, with overall dimensions of 26 × 28 × 52 Å (Fig. 4(a)). A single disulphide bond was formed between Cys42 and Cys47, linking the first β-strand to the first α-helix at the N-terminal cap structure. The right-handed β-helix fold is commonly found in carbohydrate-active enzymes, such as pectate lyases, belonging to PL1, PL3, and PL9; polygalacturonases, classified as glycoside hydrolase family 28 (GH28); PL6 alginate lyase, a catalytic domain of GH136 lacto-*N*-biosidase (LnbX); and mannuronan C-5 epimerase^11^. The number of coils, for example, 10 coils for PsAly, vary among these β-helix folds; pectate lyase Pe11A and Pel3A have 8 coils; LnbX has 15 coils; PL6 alginate lyase consists of double right-handed β-helix fold domains, each with 12 and 8 coils (AlyGC^30^ from *Paraglaciecola chathamensis* S18K6T; PDB: 5GKD; Fig. 4(b) centre); another PL6 alginate lyase with a single right-handed β-helix fold has 12 coils (AlyF^31^ from *Vibrio* OU02; PDB: 5Z9T; Fig. 4(b) right); and mannuronan C-5 epimerase has 12 coils. Although PsAly and PL6 alginate lyases (AlyGC and AlyF) share the β-helix fold, the amino acid sequence of PsAly has a low sequence similarity to PL6 and those of uncharacterised proteins of *Paenibacillus* sp., including *P*. *alvei* (Acc. No. SYX84066), *P*. *chitinolyticus* KCCM 41400 (Acc. No. QAV19742; QAV19113), *P*. *durus* DSM 1735 (Acc. No. AIQ11428), and *P*. *lautus* E7593-69 (Acc. No. AYB44622). These AlyGC and AlyF mainly hydrolyse PG in an exolytic manner, while PsAly prefers to hydrolyse PM in an endolytic manner. The active cleft structures are also different. The cleft of PsAly is an open-ended form, while the cleft structures of AlyGC and AlyF were semi-closed forms (Fig. S5). The primary and three-dimensional structure indicate that PsAly could be classified as a novel PL family and not as PL6. The DALI^32^ server indicated that the basic fold of the PsAly structure is similar to the structure of Pel9A^33^ of *Dickeya dadantii* (*Erwinia chrysanthemi*) with 10 coils, although Pel9A is a PL9 pectate lyase, not an alginate lyase. The root mean square (rms) deviations were 2.22 Å for the superimpositioning of 216 Cα atoms of PsAly onto those of Pel9A. In the PsAly structure, 32 β-strands formed a triple β-helix structure with three distorted parallel β-sheets, named PB1, PB2, and PB3, according to the naming of those of Pel9A (Fig. 4(a)). The β-strands in the helix are connected by three turns, T1 (PB1 to PB2), T2 (PB2 to PB3), and T3 (PB3 to PB1). The long cleft from the N-terminus to the C-terminus is formed by the external surface of PB1, T1, and T3, of approximately 20 × 10 × 15 Å (Fig. 4(c)). The T1 of the fifth coil of PsAly is longer than the other T1s and forms the sidewall of the cleft. The T3 of the second, fifth, and seventh coils of PsAly are also longer than the other T3s and form the wall of the cleft. There are some differences in their N- and C-terminal β-helix fold structures, as well as in the T1 and T3 turn structures, between PsAly and Pel9A; the overall structure of PsAly is similar but slightly smaller than that of Pel9A (Fig. S6). At the N-terminus, the loop between the first β-strand and the α-helix of Pel9A is longer than that of PsAly. The C-terminal region of PsAly is wound up by the last PB3 strand of the 10th coil; however, in the Pel9A structure, an extra loop extends from the last PB3 strand. This extra loop in Pel9A interacts with the outer face, formed by T3 and PB1, of the 4th to the 10th coils. The sidewall, formed by the T3 turns of Pel9A, is higher than that of PsAly (Fig. S6).

**Table 1:** Data collection and refinement statistics for AlgL structures.

	Native	Se-Met
Space group	*C*2	*C*2
Unit cell parameters (Å, °)	a = 125.6, b = 57.8, c = 39.7, β = 91.1	a = 125.7, b = 57.8, c = 39.9, β = 91.0
**Data collection**		
Resolution limit (last shell)^a^ (Å)	50.0–0.89 (0.91–0.89)	50.0–1.30 (1.32–1.30)
Measured reflections	883,113	465,033
Unique reflections	200,667 (9,592)	65,072 (3,486)
Redundancy	4.4 (4.5)	7.1 (7.2)
Completeness (|*I*| > σ|*I*|) (%)	92.6 (88.5)	92.8 (100.0)
<*I*/σ(*I)>*	29.5 (2.6)	56.7 (28.7)
*R*_merge_ (%)^b^	5.4 (67.2)	6.4 (11.5)
*R*_pim_ (%)^c^	2.8 (35.2)	2.6 (4.6)
CC_1/2_ of last shell (%)	82.9	99.4
Wilson *B* factor (Å^2^)	5.291	4.771
**Refinement**		
Final model	298 aa, 2 Na^+^, 433 H_2_O, one imidazole	
Resolution limit (Å)	23.357–0.890 (0.900–0.890)	
Used reflections	200,667	
Completeness (|*F*| > σ|*F*|) (%)	92.6 (88.0)	
Average *B*-factor (Å^2^)		
Protein	11.3	
Na^+^ ion	6.77	
Water	27.7	
Imidazole	26.8	
*R*-factor (%)^f^	10.6 (21.1)	
*R*_free_ (%)^g^	12.2 (21.5)	
Root-mean-square deviations		
Bond (Å)	0.012	
Angle (°)	1.309	
**Ramachandran plot (%)**		
Favoured region	98.0	
Allowed region	2.0	
Outlier region	0	

^a^Data in the highest resolution shells are presented in parentheses.

^b^*R*_merge_ = Σ_*hkl*_∑_i_|*I*_i_(hkl) − <*I*(hkl)> |/Σ_*hkl*_∑*I*_i_(hkl) × 100, where *I*_i_(hkl) is the intensity of individual reflection and <*I*(hkl)> is the mean intensity of all reflections.

^c^*R*_pim_ =  Σ_*hkl*_[1/(N − 1)]^1/2^Σ_i_|*I*_i_(hkl) − <*I*(hkl)> |/Σ_*hkl*_ Σ_i_*I*_i_(hkl) × 100, where *I*_i_(hkl) is the intensity of individual reflection and <*I*(hkl)> is the mean intensity of all reflections.

^d^CC_1/2_ is the correlation coefficient between random half-datasets.

^e^*R*-factor = ∑|*F*_o_ − *F*_c_|/∑|*F*_o_| × 100, where *F*_o_ is the observed structure factor and *F*_c_ is the calculated structure factor.

^f^*R*_free_ was calculated from 5% of the reflections selected randomly.

Inside the cleft, two cation-binding sites were formed on the sixth and seventh coils of the structure (Fig. 4(c,d)). These sodium ions were obtained from the crystallisation solution. Both ions were found to be tightly bound with a six-coordinate octahedral geometry by Glu181, Glu216, Asp219, and three water molecules at the first cation binding site, and Tyr244 (carbonyl group), Asp246, Gln276, and three water molecules at the second cation binding site, with an average distance between the cations and the six ligands of 2.4 Å (Fig. 4(d)). As judged by the coordination and CheckMyMetal^34^ (metal binding site validation server) analysis, sodium or calcium ions were appropriate to occupy these binding sites. After refinements with replacement of these ions on the sites, the *F*_o_–*F*_c_ electron density map showed strong negative peaks (Fig. S7(a)) in the presence of calcium ions on the sites, while the map in the presence of the sodium ions showed no such strong negative peaks (Fig. S7(b)). The occupied ions were expected to be sodium ions. Cation binding on the PB1 surface is typically observed in members of polysaccharide lyases with β-helix folds, such as pectate lyases and PL6 exo-type alginate lyase (Fig. 4(b)), and some of their catalytic mechanisms are dependent on these cations^11^. PsAly activity increased after the addition of cations, as described above (Fig. 2(g)).

### Conserved features of the active site

**Figure 5:**
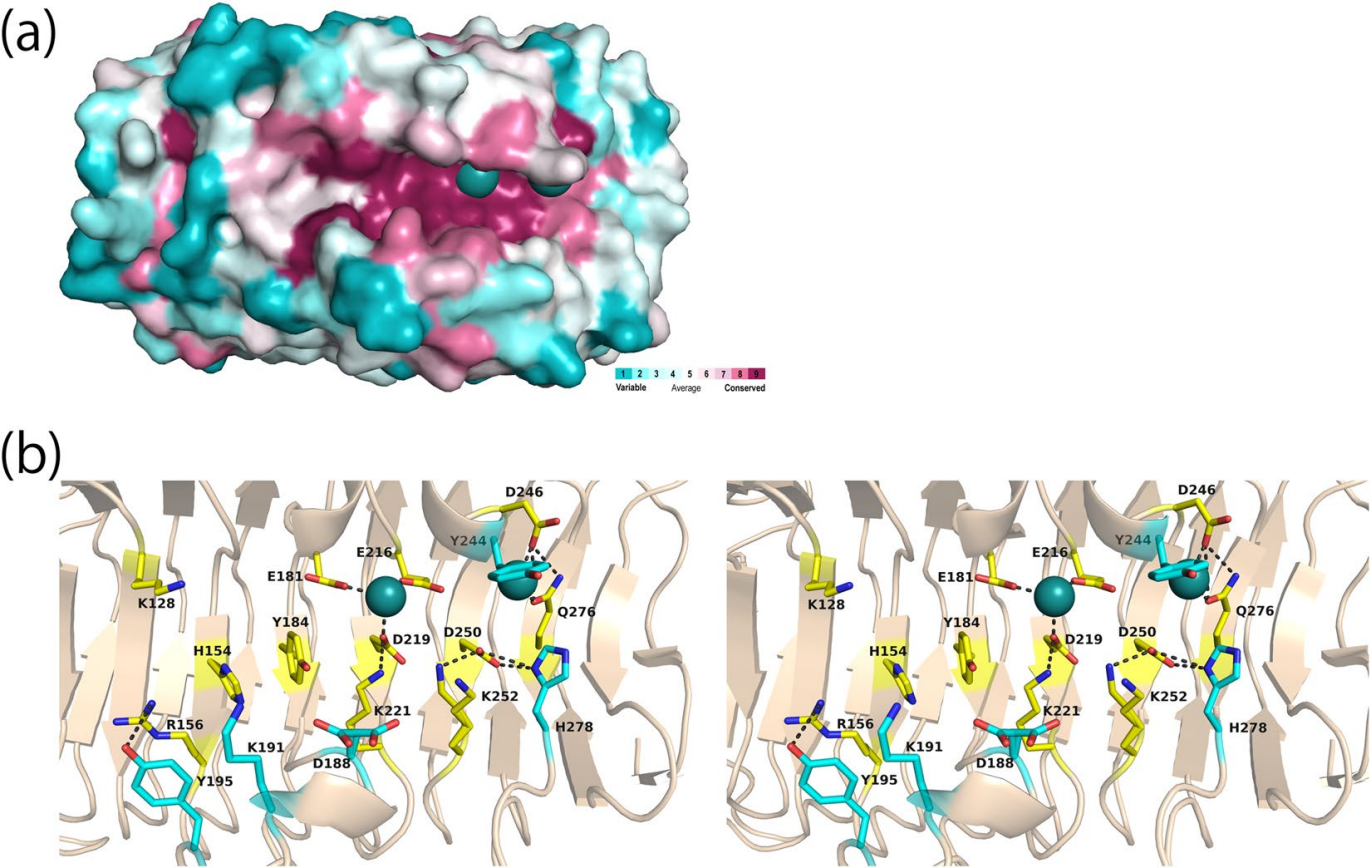
Active cleft of PsAly. (**a**) The amino acid conservation on the cleft of the PsAly. The surface of PsAly with bound sodium ions (deep teal balls) is coloured with regions of the greatest variability (cyan), modest (white) and highest conservation (magenta) using the ConSurf server. (**b**) The substrate-binding cleft is covered by the conserved residues (yellow stick models). Other ionisable residues in the cleft are also shown as cyan stick models. The blacked dashed lines represent hydrogen bond interactions (<3.5 Å).

The conservation of the amino acid residues of PsAly was analysed using the ConSurf server^35^, based on the multiple sequence alignment with 150 amino acid sequences. The amino acid sequence of PsAly showed no significant sequence identity with those of alginate lyases and any functionally characterised proteins. The sequences of interest were automatically selected from the database by the server. Almost all sequences (a.a. sequence identity, around 35–65%) were those of bacterial uncharacterised proteins (or hypothetical proteins), mainly from Gram-positive bacteria such as *Paenibacillus* sp., *Streptomyces* sp., and *Ruminococcus* sp. The conserved residues are concentrated near the cation binding sites on the cleft (Fig. 5(a)). In addition to the five residues interacting with the two cations (Fig. 4(d)), seven amino acid residues (Lys128, His154, Arg156, Tyr184, Lys221, Asp250, and Lys252) are completely conserved in the amino acid sequences on the active site (Fig. 5(b), S8). Besides these conserved residues, five ionisable residues (Asp188, Lys191, Tyr195, Tyr244, and His278) are present in the site. To investigate the importance of these residues for catalysis, the mutant enzymes (K128A, R156A, Y184F, K221A, D250N, K252A, D188N, K191A, Y195F, Y244F, and H278A) were prepared and their specific activities were measured (Fig. S9(a), Table 2). Plasmids with site-directed mutations on the residue corresponding to His154, for the design of H154A, H154S, H154G, H154D, H154N, H154E, H154Q, H154V, H154L, and H154K, could not be obtained. The circular dichroism (CD) spectra of the purified wild-type and mutant enzymes had similar profiles (Fig. S9(b–d)), indicating that the mutants did not undergo any significant conformational changes compared to the wild-type enzyme. The specific activities of all the prepared mutants were lower than those of the wild-type enzyme (Table 2). In particular, the activities of Y184F and K221A could not be determined. The residues Tyr184 and Lys221 were located near the first cation binding site. These results suggest that Tyr184 and Lys221 are significantly involved in the catalytic reaction. In addition to the conserved residues on the surface, three Asn residues (Asn261, Asn293, and Asn318) were well-conserved in all the related proteins (Fig. S8). They are orderly, stacked at T2, and packed at the core of the β-helix, which is commonly observed in the β-helix as an Asn ladder (Fig. S10).

**Table 2:** Relative activity of wild-type and mutant enzymes.

Enzyme	Relative specific activities (%)
Wild-type	100 ± 4
K128A	1.4 ± 0.2
R156A	0.79 ± 0.15
Y184F	N.D.^a^
D188N	6.8 ± 0.9
K191A	2.1 ± 0.5
Y195F	67 ± 3
K221A	N.D.
Y244F	42 ± 2
D250N	0.25 ± 0.03
K252A	0.032 ± 0.007
H278A	0.25 ± 0.01

^a^Not detected.

Recently, a novel alginate lyase, SjAly, was isolated from brown alga *Saccharina japonica* and characterised^22^. SjAly was identified as an endolytic polymannuronate lyase with a molecular mass of 39 kDa (359 amino acid residues), with a low amino acid sequence identity between PsAly and SjAly (24%) (Fig. S8). Although the two catalytically important residues (Tyr184 and Lys221) and the cation chelating residues (Glu181, Glu216, and Asp219) of the first binding site of PsAly were completely conserved in SjAly, the dependency of SjAly on cations is quite different from that of PsAly: the activity of SjAly is independent of cations and not inhibited by EDTA, whereas the activity of PsAly was enhanced by certain cations and inhibited by EDTA (Fig. 2(g)). On the other hand, the second binding site residues (Tyr244, Asp246, and Gln276) and other active site residues (His154, Arg156, Asp250, and Lys252) of PsAly are not conserved in SjAly (Fig. S8). Their final products are also different: SjAly produces a monomer to trimer from PM, whereas PsAly produced a trimer to pentamer (Fig. 3(b,c)), suggesting that the difference in their dependency on cations and the final products do not result from the catalytic mechanism, but from the difference in their active site architectures.

### Catalytic mechanism

**Figure 6:**
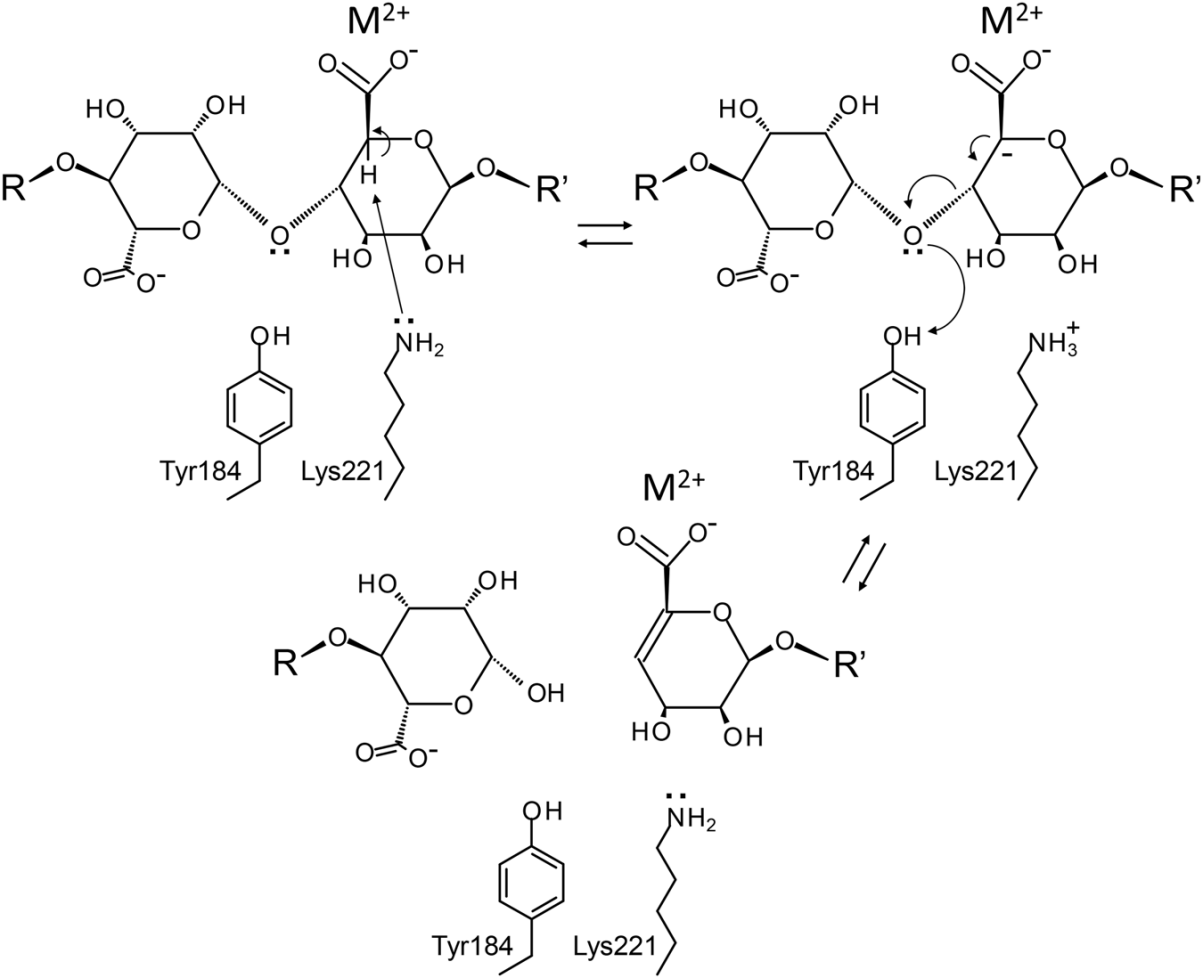
Schematic representation of the proposed catalytic mechanism of PsAly. Firstly, Lys221 acts as the general base and abstracts a proton from the C5 atom to form the carbanion intermediate. Then, Tyr184 acts as the general acid and provides a proton to the scissile glycosidic oxygen O4 from the same side (syn configuration) to release saturated and unsaturated saccharides (E1cB-elimination reaction). Cation (M^2+^) neutralises the carboxyl group of the substrate and reduces the p*K*_a_ of the C5 proton.

In general, polysaccharide lyases cleave a glyosidic bond in two steps: β-elimination with the general-base-catalysed abstraction of the proton from C5 of the substrate sugar ring, and the protonation of O4 of the glycosidic bond to release the products (E1cB-elimination reaction)^36,37^. In previously characterised polysaccharide lyases, various side-chains of amino acid residues, such as histidine, tyrosine, lysine, and arginine, have been proposed to form the Brønsted base, whereas arginine, tyrosine, and lysine residues and a water molecule have been proposed to be the acid catalyst^11,36,37^. Previous reports have also proposed that polysaccharide lyases with β-helix folds utilise a divalent cation, for example Ca^2+^, to neutralise the negative charge of the carboxyl group of the substrate and reduce the p*K*a of the C5 proton^36,37^. Indeed, the activity of PsAly was enhanced by several divalent cations and inhibited by EDTA (Fig. 2(g)). On the other hand, the catalytic mechanism is further classified to reflect the spatial arrangement of the two catalytic residues, either on the same face of the double bond (syn elimination) or on the opposite sides (anti elimination). For instance, Pel9A degrades pectate using a lysine residue as a base catalyst and a water molecule as an acid acting on the opposite side (anti elimination)^33,36^. Pectin consists of a chain of α-1,4-linked D-galacturonic acid residues. In the case of polymannuronate (PM) lyases, such as PsAly, which cleave β-1,4-glycosidic bonds, the proton of C5 and the lone pair of the scissile glycosidic oxygen O4 are arranged on the same face, such that the proton transfer reactions should occur on the same side (syn elimination). To proceed with the reaction, the saccharide binding is limited as follows: the carboxy group of the mannuronate residue should interact with the bound divalent cation; the proton of C5 and the lone pair of O4 of the glyosidic bond are positioned to face the conserved amino acid residues on the active site. Considering the position of the ligand and the mutagenic results, the sidechains of Tyr184 and/or Lys221 are the most likely candidates for the base and/or acid catalysts (Fig. 6). The optimal pH of this enzyme was around 7 to 7.5 (Fig. 2(e)). The adjacent Lys252 (Fig. 5(b)) most likely reduced the p*K*_a_ value of Lys221, in a manner similar to the p*K*_a_ shifts of the carboxylate groups in acetoacetate decarboxylase^38^. The same shift was also observed in the p*K*_a_ values of ethylene diamine (10.7 and 7.5) by the adjacent amino group.

**Figure 7:**
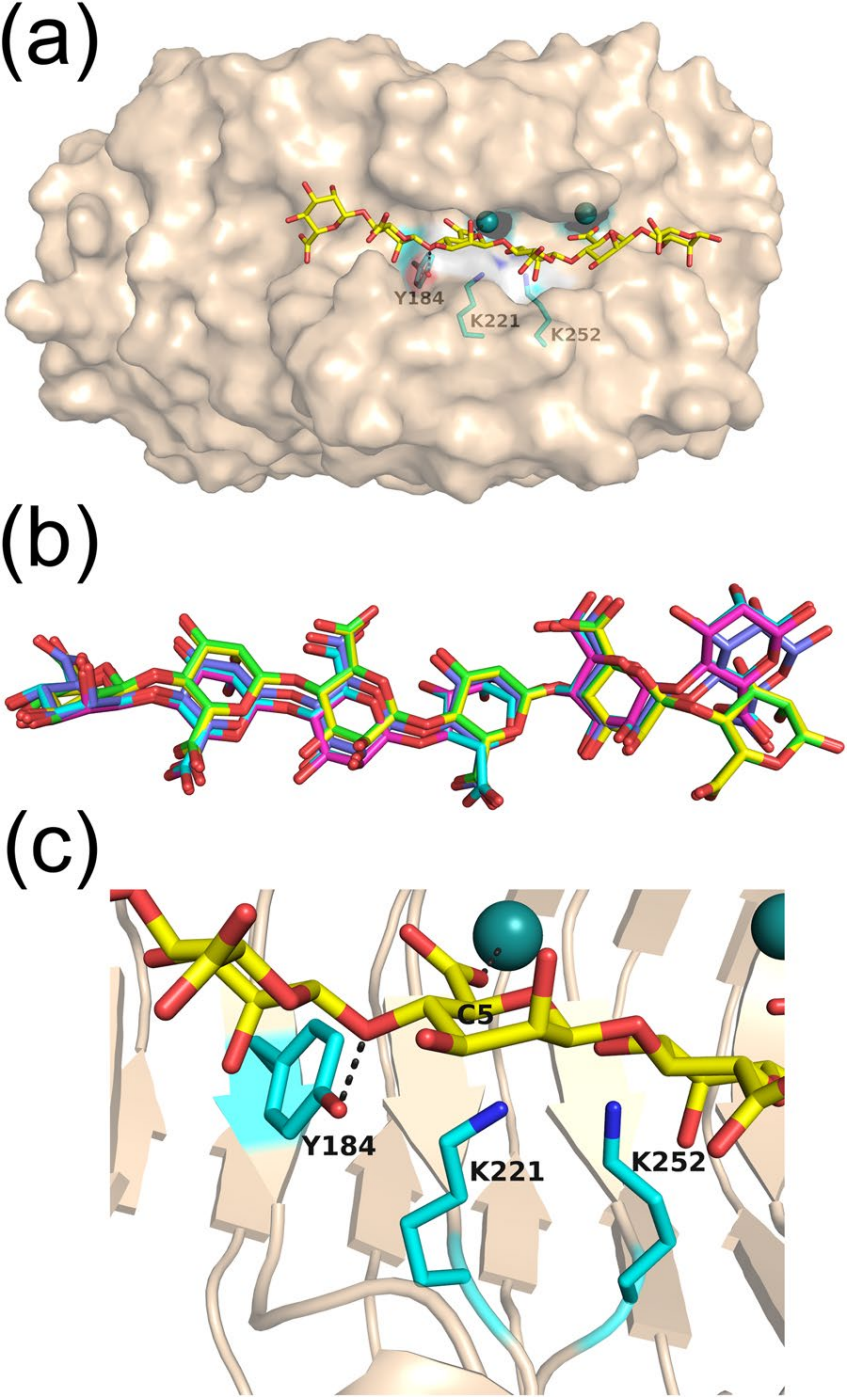
Docking simulation of PsAly with hexamannuronate (6M) using the SwissDock program. (**a**) One of the docking results with the lowest binding free energy (Δ*G* = −15.7 kcal/mol) is shown. The bound 6M model is shown as a yellow stick model. Two metal ions and important catalytic residues, Tyr184, Lys221, and Lys252, are also shown as ball and stick models (cyan). (**b**) The top five models of bound 6M ranked by the binding free energy (Table 3). The models are represented by stick models (yellow, cyan, pink, green, and violet). (**c**) Close-up view of (**a**). The hydrogen atom of C5 is close to Lys221 and the oxygen atom of glyosidic bond is close to Tyr184. Lys252 is close to Lys221.

Since it had proved difficult to prepare enzyme crystals bound with substrates or products by soaking or cocrystallisation, we resorted to carrying out molecular docking simulations using the SwissDock^39^ program to obtain the oligosaccharide binding structure. In the simulation results, hexamannuronate (6M) was bound on the bottom surface of the cleft with the lowest binding free energy (ΔG = −15.7 kcal/mol) (Fig. 7(a)), although other oligosaccharides such as dimannuronate to pentamannuronate (2M to 5M) could not be bound on the bottom surface in the attempted simulations. The top five models (Table 3) of the bound 6M ranked by the binding free energy almost had the same orientations (Fig. 7(b)). However, mannuronate residue at the reducing terminus of 6M was exposed to the solvent region (Fig. 7(a)) and the locations of this residue were different in the predicted models (Fig. 7(b)). Although multiple ligand orientations were predicted via the simulation in and around the active cleft of PsAly, two catalytically important residues, Tyr184 and Lys221, were appropriately positioned to react with the substrate in the model (Fig. 7(c)); the hydrogen atom of C5 was at a chemically relevant distance (<4.0 Å) for extraction from the ε-amino group of Lys221; the oxygen atom of the glyosidic bond was positioned close to Tyr184, within a hydrogen bond distance (<3.5 Å); the carboxy group of the mannuronate interacted with the cation (<2.4 Å). This model structure supports the hypothesis that Lys221 acts as a base catalyst and Tyr184 as an acid (Fig. 6).

**Table 3:** Docking scores of the interaction between PsAly and 6M obtained by SwissDock.

Cluster rank (model colour in Fig. 7(b))	FullFitness (kcal/mol)	Estimated ΔG (kcal/mol)
0–4 (yellow)	−688.887	−15.693207
5–7 (cyan)	−688.70404	−15.290356
8–11 (pink)	−688.538	−15.242711
12 (green)	−687.7349	−15.571159
13 (violet)	−684.38403	−14.480933

### Alginate degradation of *P*. str. FPU-7

The alginate degradation activity of the cell culture supernatant of *P*. str. FPU-7 was investigated. Alginate was found to be degraded by the supernatant of the cells at stationary phase. The activity of the supernatant was 0.031 ± 0.009 U/mg, determined using the DNS method. The Luria–Bertani (LB) medium used did not contain alginate. This suggests that the secretion of alginate-degrading enzymes is constitutive. So far, alginate lyases have been classified into 10 PL families: PL5, PL6, PL7, PL14, PL15, PL17, PL18, PL32, PL34, and PL36. The genes of these family enzymes could not be found in the draft genome of *P*. str. FPU-7. The N-terminal amino acid sequence of PsAly indicates that PsAly is a secretory enzyme. The degradation activity of *P*. str. FPU-7 may result from the constitutive secretion of PsAly.

## Methods

### Chemicals

Low viscosity sodium alginate (viscosity: 80–120 cps, 1% [w/v]) was purchased from FUJIFILM Wako Pure Chemical Corporation (Osaka, Japan). Medium and high viscosity sodium alginate (500 and 1,000 cps, 1% [w/v]) were purchased from Nacalai Tesque Inc. (Kyoto, Japan). PM, PG, and MG (DP = 20–30) were prepared from sodium alginate (1,000 cps) using Gacesa and Wusteman’s method^40^. All other chemicals and reagents were of analytical grade and purchased from FUJIFILM Wako Pure Chemical Corporation or Sigma (St. Louis, MO), unless stated otherwise.

### Primary sequence analysis

Primary sequence analysis was performed using the Pfam database^19^, BLAST^20^, and ClustalW^41^ programs. The N-terminal signal peptide of PsAly was predicted by using the SignalP 5.0 server at the Technical University of Denmark (http://www.cbs.dtu.dk/services/SignalP/)^18^.

### Cloning, expression, and purification of PsAly

The procedures for the subcloning of the *PsAly* gene and the purification of the enzymes are described in the Supplementary Information. The protein concentration was determined by UV spectrophotometry using the theoretical molar extinction coefficient ε280 = 37,485 (1/M cm) according to the ExPASy ProtParam tool server (http://web.expasy.org/protparam/)^42^.

### NH_2_-terminal amino acid sequence and gel permeation chromatography of PsAly

The procedures for the NH_2_-terminal amino acid sequence analysis and gel permeation chromatography of PsAly are described in the Supplementary Information.

### Degradation of polysaccharides by PsAly

The reaction mixtures (500 μl each) were prepared with 0.01 mg/ml purified protein, 1% (w/v) soluble or insoluble polysaccharides, such as chitin (Nacalai Tesque Inc.), chitosan (from crab shell; Nacalai Tesque Inc.), curdlan, cellulose (α-cellulose; Nacalai Tesque Inc.), carboxymethyl cellulose sodium salt (Nacalai Tesque Inc.), xylan (from beechwood; Nacalai Tesque Inc.), laminarin (Nacalai Tesque Inc.), sodium alginate (500 cps), pectin (from citrus; Nacalai Tesque Inc.), heparin sodium, xanthan gum (Nacalai Tesque Inc.), or gellan gum (Nacalai Tesque Inc.), and 50 mM Tris buffer pH 7.5. These reaction mixtures were incubated at 37 °C for 12 h. Then, the reaction mixtures were centrifuged at 15,000 *g* for 10 min and the clear fractions were transferred to clean tubes for assays. The degradation of the polysaccharides was determined using the DNS assay^43^. The DNS reagent consisted of 1% (w/v) DNS, 30% (w/v) potassium sodium tartrate, and 0.4M NaOH. The aliquots (100 μl) were mixed with equal volumes (100 μl) of DNS reagent, boiled for 5 min, cooled to 20 °C, and then observed for a red colour or measured at an absorbance of 525 nm^43^.

### Analysis of reaction products by mass spectrometry

The reactions were conducted at 37 °C for 24 h with a 100 μl solution of 50 mM Tris buffer pH 7.5, 0.4% (w/v) alginate (500 cps), and 0.01 mg/ml enzyme. After incubation, two volumes of ethanol were added to the solution. Then, the mixture was centrifuged at 15,000 *g* for 10 min and the supernatant was transferred to a tube. The concentration of the unsaturated alginate oligosaccharides was determined using the extinction coefficient of 6150 1/M cm^44^. The conditions of the samples and mass analyser (Orbitrap Elite hybrid mass spectrometer; Thermo Scientific, Waltham, MA) are described in the Supplementary Information.

### Differential scanning fluorimetry

The differential scanning fluorimetry of PsAly was performed with a SYPRO Orange Protein Gel Stain dye (5× final concentration: Thermo Fisher Scientific). The procedure and analysis for the differential scanning fluorimetry are described in the Supplementary Information.

### Alginate lyase activity assay

The alginate lyase activity of PsAly was determined by measuring the increase in absorbance at 235 nm with the formation of a carbon-carbon double bond of the product^45^. Standard assays were performed at 37 °C in a total volume of 500 μl of 50 mM Tris pH 7.5, 0.2 μM of PsAly, and 0.4% (w/v) alginate (500 cps). The thermal stability, kinetics of thermal inactivation, activity-temperature profile, activity-pH profile, and pH stability were determined by carrying out assays under various conditions. To determine the pH-activity profile, the following 50 mM buffer systems were used: sodium acetate, pH 5.5 and 6.0; imidazole, pH 6.0–7.5; and Tris, pH 7.0–9.0. To determine the pH stability, the following 50 mM buffer systems were used: glycine, pH 2.0, 3.0, 10.0, 11.0, and 11.5; sodium acetate, pH 4.0 and 5.0; sodium phosphate, pH 6.0 and 7.0; Tris, pH 8.0; *N*,*N*-bis(2-hydroxyethyl)glycine, pH 9.0. To determine the activity-temperature profile, reactions were performed at different temperatures, ranging from 4 °C to 75 °C. To determine the pH and thermal stabilities, the enzyme was preincubated for 1 h at a given pH or temperature and subjected to the standard assay. The thermal inactivation of PsAly was obtained at 37, 47, 57, and 67 °C. Aliquots were removed after incubation for 0, 5, 15, 30, and 60 min; 2, 4, 8, and 24 h (at 37, 47, and 57 °C); or 0, 0.5, 1, and 3 min (at 67 °C) and then subjected to the standard assay. The effect of cations and metal chelation on enzyme activity was determined by adding 5 mM ethylenediaminetetraacetic acid (EDTA), NaCl, KCl, MgCl_2_, MnCl_2_, CoCl_2_, NiSO_4_, CuSO_4_, Zn(O_2_CCH_3_)_2_, or 1 mM CaCl_2_ to the reactions. The kinetic parameters (*k*cat and *K*m) were determined by nonlinear fitting to the Michaelis–Menten equation with alginate (500 cps) in the range of 0.025%–0.4% (w/v), and the activity was measured as described above. The substrate specificity of PsAly was evaluated using 0.4% (w/v) alginate (1,000, 500, or 120 cps), PM, PG, or MG as the substrate.

### Analysis of reaction mode of PsAly

The reaction mixture of PsAly and 0.4% (w/v) alginate (500 cps) was monitored by an Ostwald viscometer (No. 3; Shibata Scientific Technology Ltd., Soka, Saitama, Japan). The reaction products of PsAly with 0.4% (w/v) alginate (500 cps), PM, or PG were also analysed by thin layer chromatography (TLC) (E. Merck, Darmstadt, Germany). These procedures are described in the Supplementary Information.

### Crystallisation and X-ray diffraction

An initial screening for the crystallisation of PsAly is described in the Supplementary Information. The crystallisation of PsAly was performed using the sitting-drop vapor-diffusion method at 20 °C. The drop (4 μl) consisted of 2 μl of protein solution (1 mg/ml) and 2 μl of reservoir solution (0.5 ml) containing 30% (w/v) PEG8000 and 0.1M imidazole pH 6.5. For cryoprotection, a protein crystal was soaked in the reservoir solution containing 30% (v/v) PEG400. X-ray diffraction images were acquired for the native and Se-Met derivative crystals at −173 °C under a nitrogen gas stream with a MAR MX 225HS or 225HE detector (Rayonix, L.L.C., Evanston, IL) and synchrotron radiation (λ = 0.85 or 1.0 Å for the native crystal or 0.9790 Å for the Se-Met derivative crystal) at the BL-26B1, BL-26B2, or BL-38B1 stations of SPring-8 (Japan), integrated and scaled using HKL-2000^46^ (Table 1).

### Structure determination and refinement

Phase determination and initial model building were performed using the processed Se-Met anomalous dataset (Table 1) and the SHELX(CDE)^47^ programs implemented in the HKL2MAP program package. The partial model obtained was manually rebuilt using the Coot ver. 0.8 program. Then, the model was refined using the Phenix.refine program in the PHENIX package^48^ against the native dataset (Table 1). Several rounds of refinements, followed by manual model building, were carried out to improve the quality of the model by increasing the data to a resolution of 0.89 Å. Water molecules were automatically incorporated where the *F*_o_–*F*_c_ and 2*F*_o_–*F*_c_ electron density map showed a density greater than 3.0 and 1.0 σ, respectively. Structural similarity was searched for using the Protein Data Bank (PDB)^49^ and the DALI program^32^. Structural alignments were conducted by superimposition using a fitting program in Coot. The conservation of the amino acid sequence on the surface of the crystal structure was estimated using the ConSurf server^35^. Multiple sequence alignment (150 sequences) was automatically built using the UNIREF90 database by the ConSurf server. The bound metal ions were analysed using CheckMyMetal^34^ (https://csgid.org/metal_sites). Structural figures were prepared by PyMol (DeLano Scientific, Palo Alto, CA).

### Docking simulation

Docking simulations were performed using the SwissDock server^39^. The crystal structure of PsAly was used as the target molecule and oligosaccharides, such as dimannuronate to hexamannuronate (2M to 6M), were used as the ligand. The structure of 6M was built by linking two trimannuronate molecules from the crystal structure of alginate ABC transporter complexed with unsaturated tetramannuronate^50^ (PDB: 4XIG), followed by energy minimisation with AutoDock version 4.2^51^ software. The other oligosaccharide models (2M to 5M) were prepared from the model of 6M.

### Site-directed mutagenesis and CD spectra measurement

The residues Lys128, Arg156, Tyr184, Asp188, Lys191, Tyr195, Lys221, Tyr244, Asp250, Lys252, and His278 in PsAly were replaced with an alanine, an asparagine, or a phenyl alanine residue, such as K128A, R156A, Y184F, D188N, K191A, Y195F, K221A, Y244F, D250N, K252A, and H278A, using site-directed mutagenesis with the expression plasmid for PsAly and the appropriate primers (Table S3). The mutagenesis, the evaluation of the purity of the enzymes, and the structural conformations of the enzymes by far-UV CD spectroscopy are described in the Supplementary Information.

### Alginate degradation activity of the culture supernatant of P. str. FPU-7

The measurement of the alginate degradation activity of the culture supernatant of *P*. str. FPU-7 is described in the Supplementary Information.

### Nucleotide sequence

The nucleotide sequence of the *aly* gene in *P*. str. FPU-7 has been deposited in DDBJ/EMBL/GenBank database under accession number LC490364.

### Protein Data Bank

The coordinates and structure of PsAly are available in the Protein Data Bank (PDB) under accession number 6KFN.

## Supplementary information

Supporting information
