# Supplementary material for: Structural and biochemical characterisation of a novel alginate lyase from Paenibacillus sp. str. FPU-7
Source: Sci Rep. 2019 Oct 16;9:14870. doi: 10.1038/s41598-019-51006-1 (PMC6796002; doi:10.1038/s41598-019-51006-1)
Supplement: Supplementary file 1 — Supporting information [file 41598_2019_51006_MOESM1_ESM.pdf]

**Supplementary Information**

# Structural and biochemical characterisation of a novel alginate lyase from *Paenibacillus* sp. str. FPU-7

Takafumi Itoh[1, *], Emi Nakagawa[1], Moe Yoda[1], Akari Nakaichi[1], Takao Hibi[1], and Hisashi Kimoto[1]

[1]Department of Bioscience and Biotechnology, Fukui Prefectural University, 4-1-1 Matsuoka Kenjyoujima, Eiheiji-cho, Yoshida-gun, Fukui 910-1142, Japan.

*Correspondence and requests for materials should be addressed to T.I. (e-mail: ito-t@fpu.ac.jp)

**Contents**

Methods

Table S1. Characteristic peaks in negative-ESI MS spectra of the reaction product of PsAly

Table S2. Specific activities of PsAly and other alginate lyases

Table S3. Primers used in cloning and site-directed mutagenesis

Figure S1. Schematic presentation of the genetic organisation of PsAly and neighbouring genes

Figure S2. Visualisation of the degradation activity of PsAly against several polysaccharides

Figure S3. Fragmentation spectra (negative-ESI MS/MS) of the $[M-H]^-$ ion at m/z 527.09 (a), m/z 703.12 (b), and m/z 879.16 (c) in the enzymatic reaction sample of PsAly

Figure S4. Kinetics of the thermal inactivation of PsAly

Figure. S5. The structural comparisons between PsAly (left) and PL6 alginate lyases (AlyGC [centre] and AlyF [right])

Figure S6. Structural comparison of PsAly and Pel9A

Figure S7. $F_o$–$F_c$ and $2F_o$–$F_c$ maps around the two bound metals

Figure S8. Amino acid sequence alignment of PsAly and other homologous proteins using the ClustalW program

Figure S9. SDS-PAGE profile (a) and far-UV circular dichroism (CD) spectra (b–d) of PsAly wild-type and mutant enzymes

Figure S10. The conserved amino acid residues in the inner structure of PsAly

Full wwPDB X-ray Structure Validation Report (PDB: 6KFN)

## Methods

### *Cloning, expression, and purification of PsAly*

The coding sequence of the mature PsAly (Ala36 to Asn317) was amplified by polymerase chain reaction (PCR) using KOD -Plus- Neo polymerase (Toyobo, Osaka, Japan), a single colony of *P*. str. FPU-7 as the template, and synthetic oligonucleotides with *Nde*I and *Xho*I restriction sites added to their termini as the forward and reverse primers (Table S3). The PCR product was ligated to pET21b (Novagen, WI) in frame to a six-histidine tag at the C terminus using the In-fusion HD Cloning Kit (Takara Bio, Kusatsu, Japan). Successful plasmid construction was confirmed by DNA sequencing, using an ABI PRISM 3130xl Genetic Analyzer (Applied Biosystems, Foster City, CA). The expression vector was transformed into competent *E. coli* BL21(DE3) cells (Novagen) or T7 Express Crystal cells (New England Biolabs Inc., Ipswich, MA). The transformant of *E. coli* BL21(DE3) was grown in 0.5 l of Luria–Bertani (LB) medium containing 50 μg/ml ampicillin at 37°C. For the expression of a PsAly derivative with selenomethionine (Se-Met), *E. coli* cells were aerobically cultured in a minimal medium supplemented with 25 μg/ml of Se-Met. When the turbidity at 600 nm reached 0.6, the cultures were supplemented with 1.0 mM isopropyl-β-D-thiogalactopyranoside, followed by further incubation at 20°C for 20 h. The cultured cells were collected by centrifugation at 6,000 *g* and 4°C for 5 min. The harvested cells were ultrasonically disrupted in 20 mM sodium phosphate buffer at pH 7.4 and 0.1 mM phenylmethylsulphonyl fluoride. The clear solutions were obtained after centrifugation at 15,000 *g* at 4°C for 20 min. Then, the proteins were fractionated with $(NH_4)_2SO_4$ from 35% to 70% saturation. The precipitants were dissolved in 20 mM sodium phosphate buffer pH 7.4, containing 20 mM imidazole and applied to an Ni-immobilised metal affinity column (Ni-IMAC; HisTrap HP 5 ml column; GE Healthcare, Little Chalfont, UK). The column-absorbed proteins were eluted with a linear gradient of imidazole (0.02-0.15 M) in 20 mM sodium phosphate buffer pH 7.4 (50 ml). The solution containing PsAly was dialysed at 277 K overnight against 10 mM tris(hydroxymethyl)aminomethane (Tris) buffer

pH 7.5. The protein was further purified by anion exchange chromatography (AEC) using a HiTrap Q HP 5 ml column (GE Healthcare) equilibrated with 20 mM Tris buffer pH 7.5 and eluted with a liner gradient of 0–0.15 M NaCl in the same buffer. The eluted proteins were dialysed at 277 K overnight against 10 mM Tris buffer pH 7.5 and used as a purified protein source. The protein concentration was determined by UV spectrophotometry using the theoretical molar extinction coefficient ε280 = 37,485 (1/M cm) according to the ExPASy ProtParam tool server (http://web.expasy.org/protparam/)[1]. The proteins were purified from the bacterial cells to homogeneity, as assessed by 15% (w/v) sodium dodecyl sulphate-polyacrylamide gel electrophoresis (SDS-PAGE) followed by Coomassie Brilliant Blue (CBB) R-250 staining.

### *$NH_2$-terminal amino acid sequence and gel permeation chromatography of PsAly*

The purified proteins (100 pmol) were adsorbed onto PVDF membranes using ProSorb Sample Preparation Cartridges (Applied Biosystems, Foster City, CA). Protein sequencing was conducted on each membrane by automated Edman degradation using a PE Applied Biosystems ‘model 491’ Procise protein sequencing system (Applied Biosystems).

The molecular mass of PsAly was evaluated by gel permeation chromatography using Superdex 200 GL 10/300 (24 ml; GE Healthcare) equilibrated with 20 mM Tris pH 7.5 containing 0.15 M sodium chloride. The molecular mass of the enzyme was calculated by the following external standards: blue dextran (molecular mass = 2,000 kDa, GE Healthcare), horse spleen ferritin (440 kDa), bovine serum albumin (dimer, 134 kDa; monomer, 67 kDa), pigeon cytochrome C (12 kDa), cyanocobalamin (1355 Da), and vitamin B2 (376 Da).

### *Analysis of reaction products by mass spectrometry*

Samples for mass analysis (negative-ESI MS and MS/MS) were prepared by adding 5–10 pmol/μl of sample to 0.5 mM $NH_4HCO_3$ and 50% (v/v) acetonitrile. The diluted samples were introduced by direct infusion (5 μl/min) into the electrospray ionisation source of the Orbitrap Elite Hybrid Mass Spectrometer (Thermo Scientific), operating in negative mode. The electrospray capillary was held at 4.5 kV and the transfer tube was held at 200°C. The precursor ions were fragmented in the ion trap by collision-induced dissociation with the appropriate collision energy.

***Differential scanning fluorimetry***

The differential scanning fluorimetry assay mixtures contained 20 mM Tris pH 7.5, 0.25 mg/ml PsAly, and SYPRO Orange Protein Gel Stain dye (5 × final concentration: Thermo Fisher Scientific) in a total volume of 20 μl. The melting curves (increase in fluorescence) were monitored by a real-time PCR instrument (StepOne; Thermo Fisher Scientific) using a ROX filter. The samples were heated from 25 to 95°C at a constant rate of 1°C/min. Melting temperatures ($T_m$) were calculated as an inflection point of the melting curve, assuming a two-state unfolding model, using Protein Thermal Shift software (TmD in the software; Thermo Fisher Scientific). The assays were performed in triplicate.

***Site-directed mutagenesis and circular dichroism (CD) spectra measurement***

The residues Lys128, Arg156, Tyr184, Asp188, Lys191, Tyr195, Lys221, Tyr244, Asp250, Lys252, and His278 in PsAly were replaced with an alanine, asparagine, or phenyl alanine, such as K128A, R156A, Y184F, D188N, K191A, Y195F, K221A, Y244F, D250N, K252A, and H278A, using a QuikChange Multi Site-Directed Mutagenesis Kit (Agilent, CA) or a KOD -Plus- Mutagenesis Kit (Toyobo) with an expression plasmid for PsAly and the appropriate primers (Table S3). The mutations were confirmed by DNA sequencing. *E. coli* BL21(DE3) cells were transformed with these mutant

plasmids, and the mutant enzymes were expressed and purified using a procedure similar to that used for the wild-type enzyme. Enzyme purity was assessed by SDS-PAGE followed by CBB R-250 staining. The specific activities were determined by the standard assay described in the main text. The wild-type and mutant enzymes were dialysed against 10 mM sodium phosphate buffer pH 7.4 to evaluate the structural conformations by far-UV CD spectroscopy using a Jasco J805 Spectropolarimeter at 205–250 nm with a demountable quartz cell with a 0.1 mm path length.

***Analysis of reaction mode of PsAly***

The reaction mixture containing 100 ml of 50 mM Tris pH 7.5, 0.2 μM of PsAly, and 0.4% (w/v) sodium alginate (500 cps) was incubated at 37°C for 0, 0.5, 1, 2, 4, and 24 h. Aliquots (10 ml each) were removed at intervals and subjected to viscosity measurement on an Ostwald viscometer (No. 3; Shibata Scientific Technology LTD., Soka, Saitama, Japan). The reaction products (5 μl, 20 μg) of PsAly with 0.4% (w/v) sodium alginate (500 cps), PM, or PG were also analysed by thin layer chromatography (TLC) (E. Merck, Darmstadt, Germany) with a solvent system of 1-butanol:formic acid:water (4:6:1, v/v). The oligosaccharide products were visualised by heating TLC plates at 130°C for 5 min after spraying with 10% (v/v) sulphuric acid in ethanol.

***Initial screening for the crystallisation***

The initial screening for the crystallisation of PsAly was performed using the commercially available crystallisation kits, Crystal Screen 1 and 2 (Hampton Research, Aliso Viejo, CA) and the Wizard Classic Crystallization Screens 1 and 2 (Emerald BioSystems, Bedford, MA) with the sitting-drop vapor-diffusion method in 96-well plates at 20°C. The crystallisation conditions were further refined using a 24-well plate and performed using the sitting-drop vapor-diffusion method at 20°C.

***Alginate degradation activity of the culture supernatant of P. str. FPU-7***

To measure the specific activity of the cell culture supernatant of *P*. str. FPU-7 against alginate, *P*. str. FPU-7 was grown in 2 ml of LB at 30°C. When the turbidity at 660 nm reached 0.8, the culture supernatants were collected by centrifugation at 12,000 *g* and 4°C for 10 min and filtered (0.45 μm syringe filter; Merck Millipore, Burlington, MA). The reactions were conducted at 37°C for 24 h with a solution of 50 mM Tris buffer pH 7.5, 0.4% (w/v) sodium alginate (500 cps), and 200 μl of the supernatant (total 500 μl). The liberated reducing ends of the aliquots (100 μl) at 0, 8, and 24 h were measured using the DNS method, as described in the main text. The standard curve was prepared using glucuronic acid. The protein concentration of the culture supernatant was determined using the Bradford Protein Assay Kit (Bio-Rad Laboratories, Inc., Hercules, CA) with bovine serum albumin as the standard.

**References**

1 Gasteiger, E. *et al.* ExPASy: The proteomics server for in-depth protein knowledge and analysis. *Nucleic Acids Res.* **31**, 3784–3788; doi:10.1093/nar/gkg563 (2003).

**Supplementary Tables**

**Table S1. Characteristic peaks in negative-ESI MS spectra of the reaction product of PsAly**

| Compound (formula) | Ion assignment | Theoretical (*m*/*z*) | Observed (*m*/*z*) |
|---|---|---|---|
| dDP3 ($C_{18}H_{24}O_{18}$) | $[M-H]^-$ | 527.09 | 527.09 |
| dDP4 ($C_{24}H_{32}O_{24}$) | $[M-H]^-$ | 703.12 | 703.12 |
| | $[M-2H]^{2-}$ | 351.06 | 351.06 |
| dDP5 ($C_{30}H_{40}O_{30}$) | $[M-H]^-$ | 879.15 | 879.16 |
| | $[M-2H]^{2-}$ | 439.07 | 439.07 |
| dDP6 ($C_{36}H_{48}O_{36}$) | $[M-H]^-$ | 1055.18 | 1055.19 |
| dDP7 ($C_{42}H_{56}O_{42}$) | $[M-2H]^{2-}$ | 615.10 | 615.11 |

**Table S2. Specific activities of PsAly and other alginate lyases**

| Enzyme[Ref.] | Source | PL family | Specific activity (substrate) (U/mg)[a] | Specific activity (substrate) (U/mg)[b] | Optimal pH | Substrate specificity |
|---|---|---|---|---|---|---|
| PsAly | *P*. str. FPU-7 | | 18 (alginate) | 2,200 (alginate) | 7.0–7.5 | PM |
| SjAly[22] | *Saccharina japonica* | | 14 (PM) | | 8.0 | PM |
| A1-III[23] | *Sphingomonas* sp. A1 | 5 | | 450 (alginate) | 7.2 | PM |
| FsAlyPL6[24] | *Flammeovirga* sp. NJ-04 | 6 | | 20 (PG), 220 (PM), 480 (alginate) | 9.0 | PM, PG |
| A1-II[23] | *Sphingomonas* sp. A1 | 7 | | 1,090 (alginate) | 7.2 | PG |
| HdAly[25] | *Haliotis discus hannai* | 14 | | 380 (alginate), 1,800 (PM) | 7.0 | PM |
| A1-IV[14] | *Sphingomonas* sp. A1 | 15 | 0.12 (tetrasaccharide) | | 7.5 | PM, PG |
| Alg17B[26] | *Saccharophagus degradans* 2-40 | 17 | | 400 (alginate) | 7.5 | PM, PG |
| Aly-SJ02[27] | *Pseudoalteromonas* sp. SM0524 | 18 | | 3,100 (PG), 4,200 (PM), 4,800 (alginate) | 8.5 | PM, PG |

[a] One unit (U) was defined as the amount of enzyme required for the transformation of 1 μmol of substrate per min.

[b] One unit (U) was defined as the amount of enzyme required to increase the absorbance at 235 nm by 0.1 per min.

**Table S3. Primers used in cloning and site-directed mutagenesis**

Underlined sequences indicate the restriction enzyme sites (*Nde*I or *Xho*I).

Bold indicates the mutated sequences.

| Construct name | Primer sequence (5′ to 3′) |
|---|---|
| Wild-type | GGAGATATACATATGGCAACCCGGACGATCTCC |
| | GTGGTGGTGCTCGAGGTTGGCGGGCGCAGCGTTTAC |
| K128A | GACCGGGGCCTTA**GC**GGGCATTATGCTC |
| | GAGCATAATGCCC**GC**TAAGGCCCCGGTC |
| R156A | GCGTCCACTTC**GCA**GACGGCAGCTC |
| | GAGCTGCCGTC**TGC**GAAGTGGACGC |
| Y184F | TTTTGGCGAAGGCATC**TTT**GTCGGCTCGGATAAAG |
| | CTTTATCCGAGCCGAC**AAA**GATGCCTTCGCCAAAA |
| D188N | GCATCTATGTCGGCTCG**AAT**AAAGGCAAATGGGC |
| | GCCCATTTGCCTTT**ATT**CGAGCCGACATAGATGC |
| K191A | GGCTCGGATAAAG**GCG**CGTGGGCTACCTATAAC |
| | GTTATAGGTAGCCCA**CGC**GCCTTTATCCGAGCC |
| Y195F | CAAATGGGCTACCT**T**TAACAAGTCCGCCG |
| | CGGCGGACTTGTTA**A**AGGTAGCCCATTTG |

| | |
|---|---|
| K221A | CGAGCATATTGATATT**GCG**GAAGGTACGGTAGGC |
| | GCCTACCGTACCTTC**CGC**AATATCAATATGCTCG |
| Y244F | CATTACGGGAGCCAACT**T**TGCCGACAGCTTTATC |
| | GATAAAGCTGTCGGCA**A**AGTTGGCTCCCGTAATG |
| D250N[a] | **AAT**GTCAAAGGCAACGACGCCGT |
| | GATAAAGCTGTCGGCATAGT |
| K252A | CAGCTTTATCGACGTC**GCG**GGCAACGACGCCGTG |
| | CACGGCGTCGTTGCC**CGC**GACGTCGATAAAGCTG |
| H278A[a] | **GC**TGTTCAGGTCGCAGGCTGGG |
| | CACTTGAAACGCATCGACAA |

[a]These mutants were prepared by using the KOD -Plus- Mutagenesis Kit. The other mutants were prepared by using the QuikChange Multi Site-Directed Mutagenesis Kit.

## Supplementary figure legends

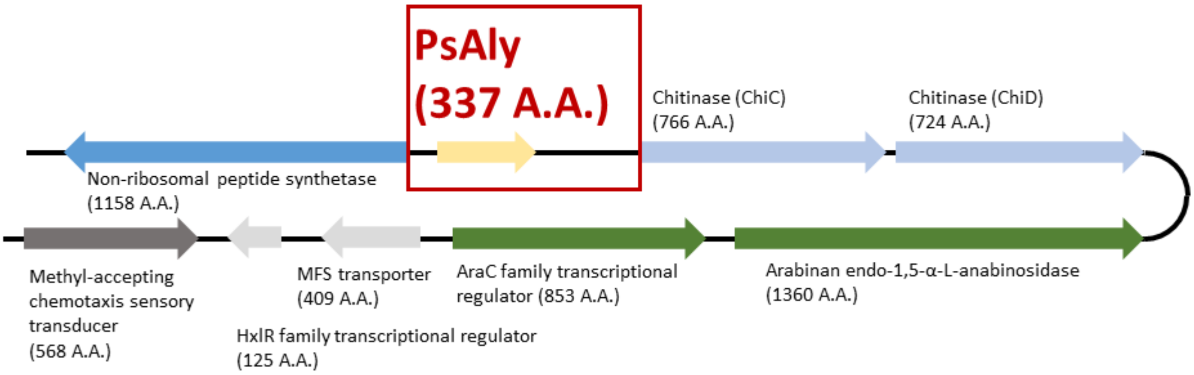


**Figure S1. Schematic presentation of the genetic organisation of PsAly and neighbouring genes**

The gene of PsAly (1,014 bp in length) was found beside two chitinase genes, *ChiC* and *ChiD*. Other neighbouring genes were assigned by the amino acid sequence homologies with a BLAST database search.

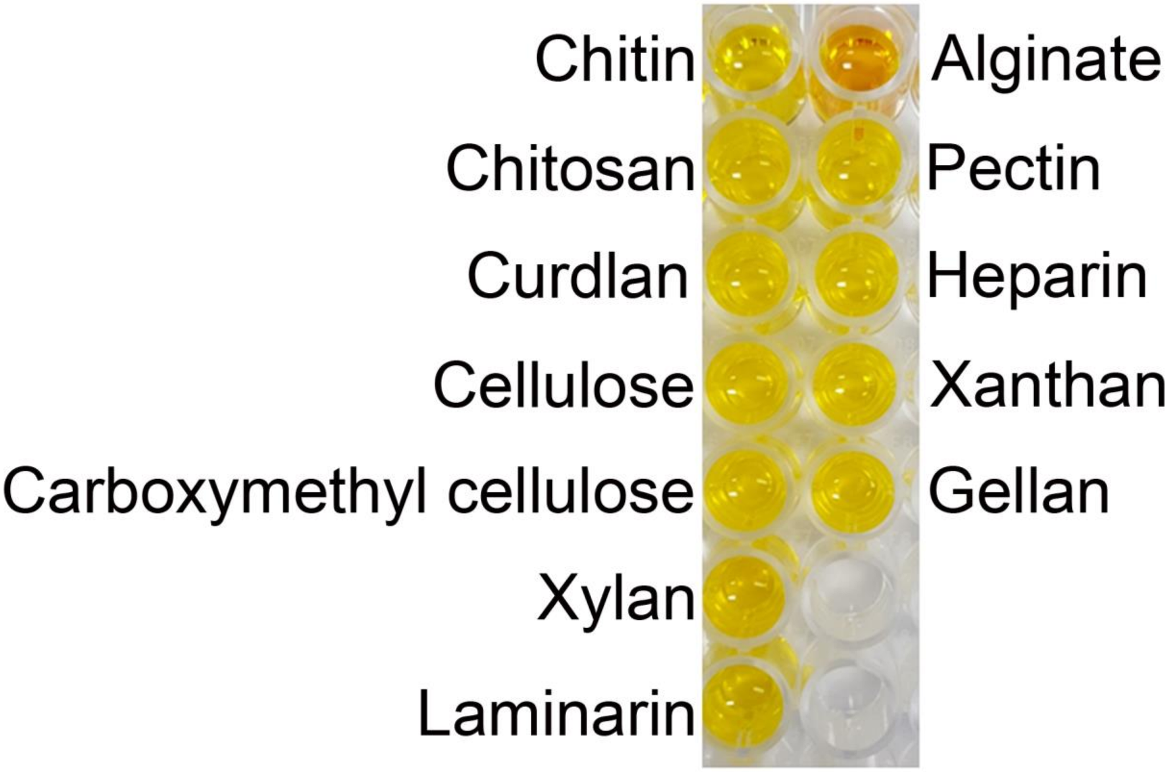


**Figure S2. Visualisation of the degradation activity of PsAly against several polysaccharides**

A mixture of several polysaccharides (chitin, chitosan, curdlan, cellulose, carboxymethyl cellulose, xylan, laminarin, alginate, pectin, heparin, xanthan, and gellan) and PsAly were incubated at 37°C for 12 h. The incubated solution was mixed with equal volumes of DNS reagent and boiled for 5 min. PsAly exhibited degradation activity for alginate, indicated by a red colour.

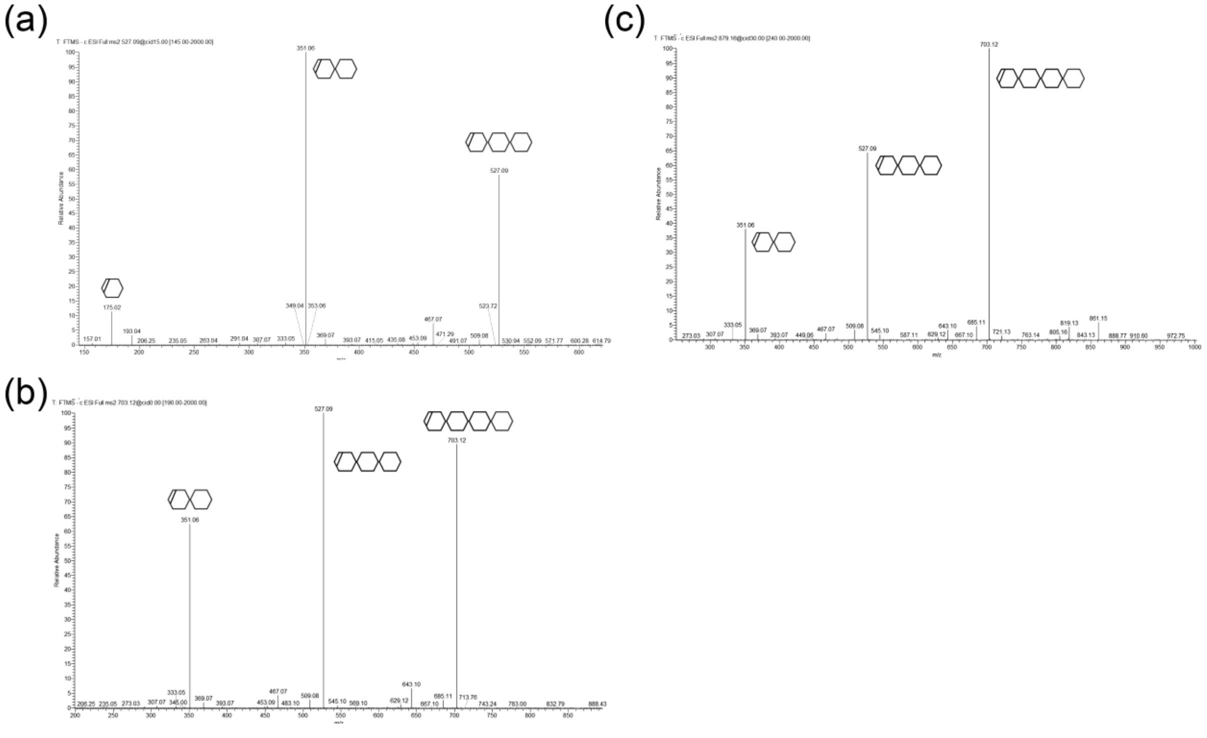


**Figure S3. Fragmentation spectra (negative-ESI MS/MS) of the [M-H]$^-$ ion at m/z 527.09 (a), m/z 703.12 (b), and m/z 879.16 (c) in the reaction sample of PsAly and alginate**

Fragment ions were observed in the MS/MS spectra after the cleavage of the glycosidic bonds by collision-induced dissociation. (a) The parent ion at m/z 527.09 corresponds to the [M-H]$^-$ ion of dDP3 ($[C_{18}H_{24}O_{18}]^-$; theoretical monoisotopic mass = 527.09). Two fragment ions at m/z 175.02 and 351.06 correspond to the [M-H]$^-$ ions of monomer ($[C_6H_7O_6]^-$; theoretical monoisotopic mass = 175.02) and dDP2 ($[C_{12}H_{15}O_{12}]^-$; theoretical monoisotopic mass = 351.06). (b) The parent ion at m/z 703.12 corresponds to the [M-H]$^-$ ion of dDP4 ($[C_{24}H_{31}O_{24}]^-$; theoretical monoisotopic mass = 703.12). Two fragment ions at m/z 351.06 and 527.09 correspond to the [M-H]$^-$ ions of dDP2 and dDP3. (c) The parent ion at m/z 879.16 was fragmented and could not be observed. Three fragment ions at m/z 351.06, 527.09, and 703.12 correspond to the [M-H]$^-$ ions of dDP2, dDP3, and dDP4.

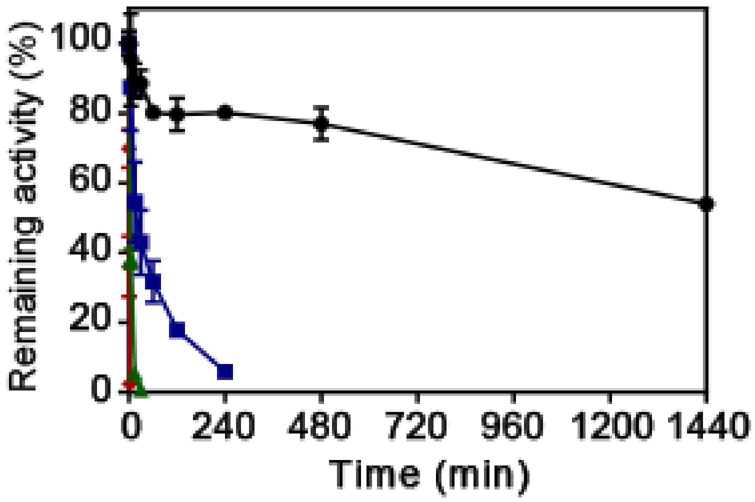


**Figure S4. Kinetics of the thermal inactivation of PsAly**

The kinetics of the thermal inactivation of PsAly at 37°C (black circle), 47°C (blue square), 57°C (green triangle), or 67°C (red diamond) was evaluated by measuring the remaining activity (%) at appropriate intervals. After incubation for 1,440 min at 37°C, the enzyme was stable at more than 50% relative activity.

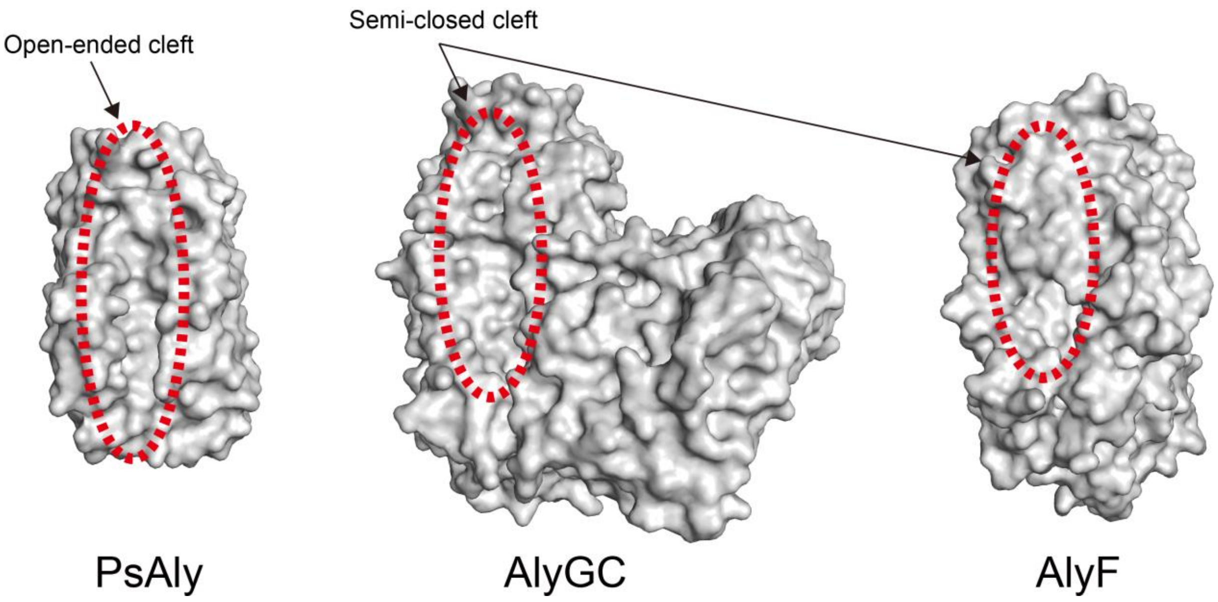


**Figure. S5. The structural comparisons between PsAly (left) and PL6 alginate lyases (AlyGC [centre]) and AlyF [right])**

Active clefts are surrounded by red circles. The cleft of PsAly is an open-ended form, while the cleft structures of PL6 alginate lyases (AlyGC and AlyF) were semi-closed forms.

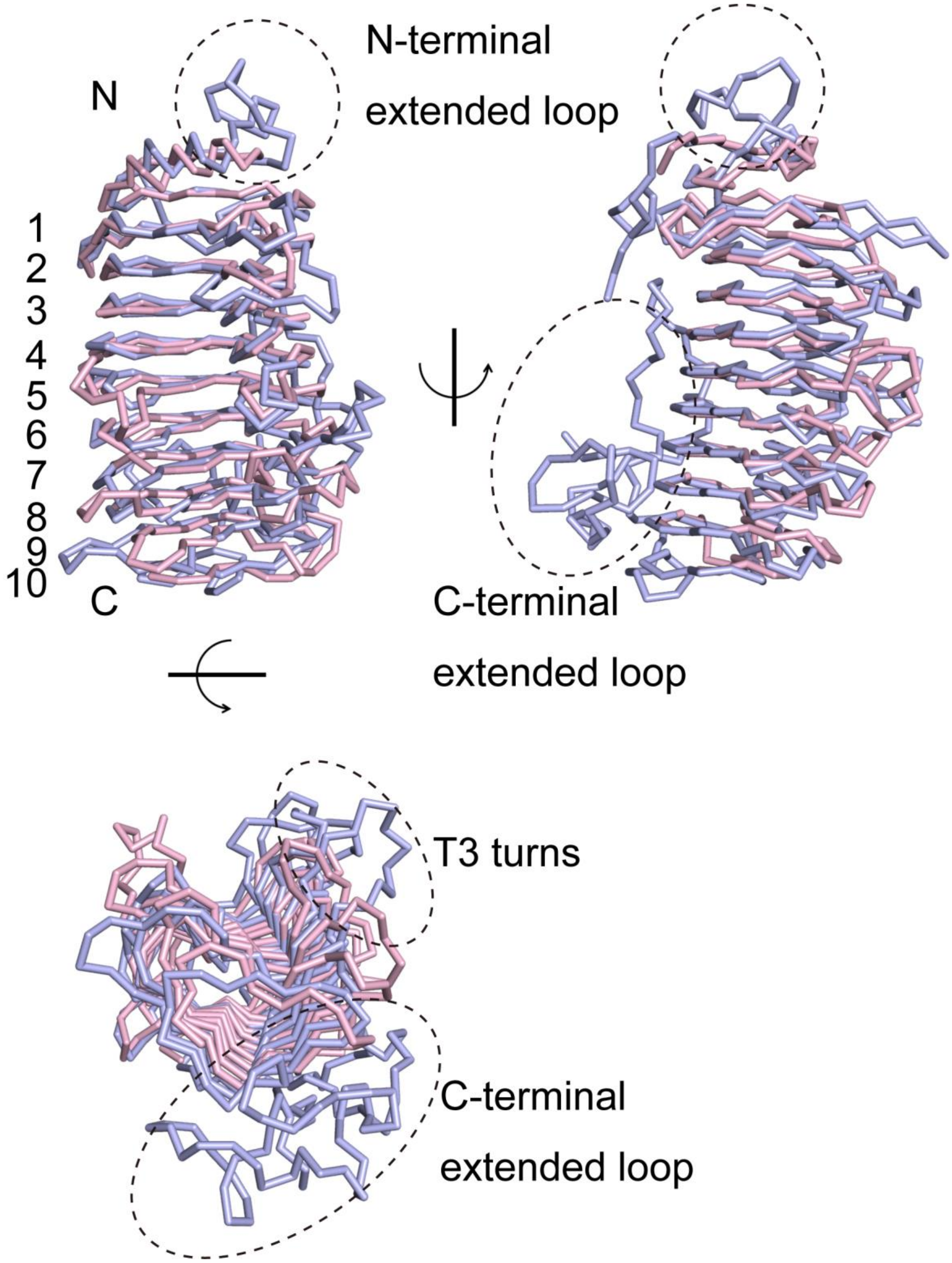


**Figure S6. Structural comparison of PsAly and Pel9A.**

The overall architecture of PsAly (pink ribbon model) is similar to that of Pel9A (blue ribbon model). Although their basic folds of 10 coils containing three parallel β-sheets superimpose well, the structure

of PsAly is slightly smaller than that of Pel9A. The loop between the first β-strand and the α-helix at N-terminus of Pel9A is longer than that of PsAly, and an extended loop is found at the C-terminus in Pel9A (black dashed circles).

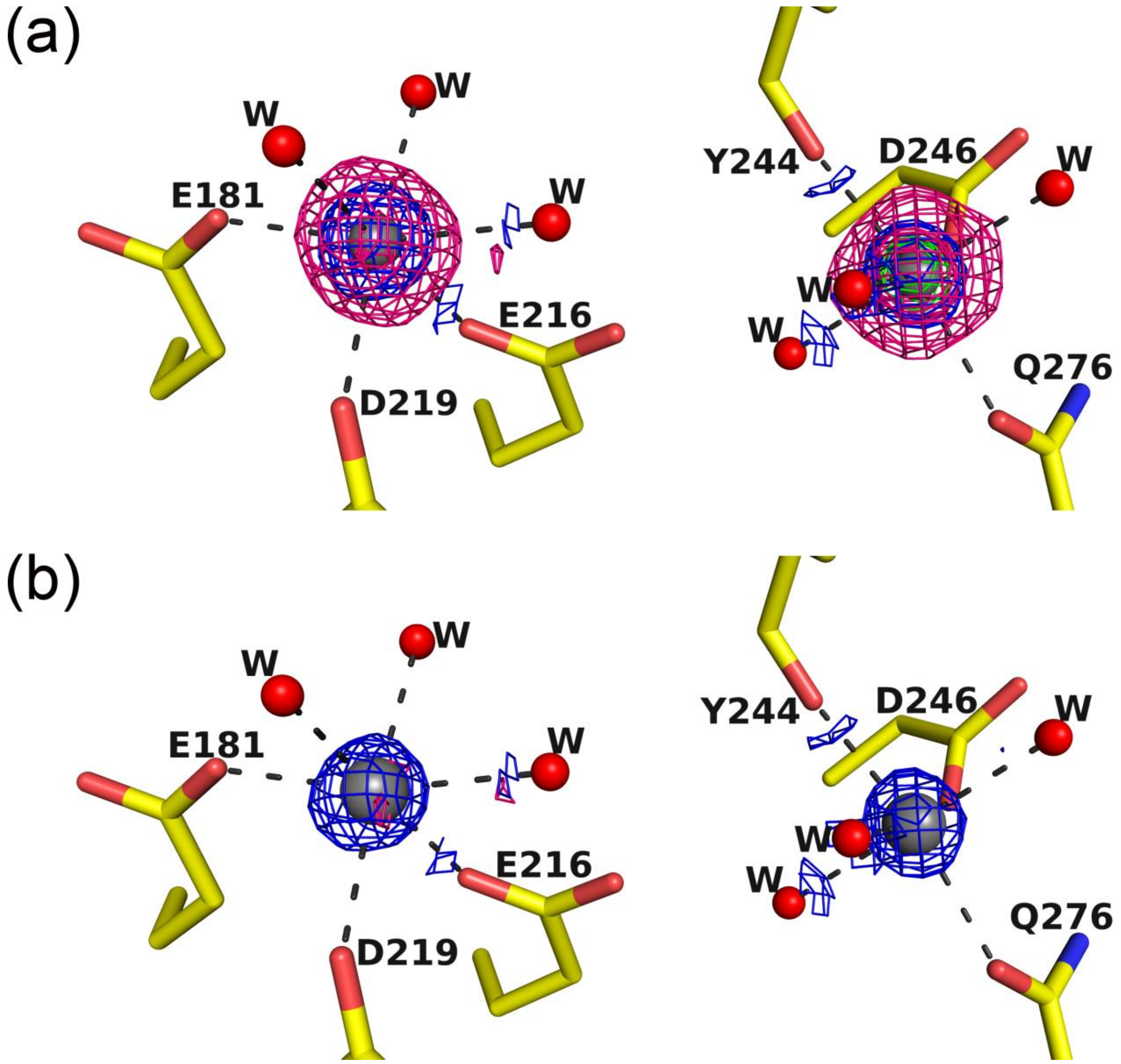


**Figure S7. $F_o$–$F_c$ and 2$F_o$–$F_c$ maps around the two bound metals**

$F_o$–$F_c$ maps are shown in red (negative) and green (positive) contoured at the ± 4.5 σ level. 2$F_o$–$F_c$ maps are shown in blue contoured at the 1.5 σ level. The amino acid residues and water molecules at the metal binding sites are represented by yellow stick and red ball models, respectively. (a) After refinements with the replacement of $Ca^{2+}$ ions (grey balls) on the sites, the $F_o$–$F_c$ electron density map showed strong negative peaks on the sites. (b) The map in the presence of $Na^{2+}$ ions (grey balls) showed no such strong negative peaks.

```
PsAly                          ------MKLLRHPASRTLAAIAAGTLCFSLFSLTTGTPGSAATR----TI 40
Paenibacillus_sp._YN15         -----------------MFKFVVL---AAMLVATSLSIPTPSQAA----SV 27
Paenibacillus_ehimensis        ---------MKKSWRSRLSCIST---VLTLGSVLLSFPASSMAA----SV 34
Paenibacillus_elgii            ---------MKKSWRSRLSCISS---VLTLGSVLLSFPASSMAA----SV 34
Paenibacillus_alvei            ----------MAVWPDFLLVISLAPTQALAWSSIAGEPLSATVRGDAKEI 40
SjAly_Saccharina_japonica      MVVSGTCLDLLLPAIIMLATLTPAVTAATTYKVTPNGKWTSSSG----YS 46
                                                :  :              .   .:

PsAly                          SCATASCLQSALKNAKPGDDIVLAEGVTFKGSFKAEASGTASQPITIRSA 90
Paenibacillus_sp._YN15         TCSTAACFTSALANAQPGDVITLAAGVTFTGNFVAAKNGTSSAKITIKGA 77
Paenibacillus_ehimensis        TCSTAACLKNALANASPGDVITLAAGVTFNGNFVAAANGTSTGKITLQSA 84
Paenibacillus_elgii            TCSTAACLKNALANASPGDVITLEAGVTFNGNFVAAANGTSTGKITLQSA 84
Paenibacillus_alvei            TCNTTSCLSKALKRITPGSSIVLAPGI-YKGSFSSDINGAFGQPIVIRSA 89
SjAly_Saccharina_japonica      SSSKIYSLPGALAIAKAGDTILLADGT-YTDRLESYRDGKSSSPIKIKGG 95
                               :. .  .:  **    .*. * *  *  :.. : :  .*     * ::..

PsAly                          GSVNP-------------AVLSGYSTGGGYSLYVTGDYWNITGLKMTGAL 127
Paenibacillus_sp._YN15         SSTNK-------------PILNGGTTSSGYALYITGDYYDVRNVKITNAK 114
Paenibacillus_ehimensis        SSSNK-------------AELNGGGTGSGYALHVTGDHWVIKDLKVTNAK 121
Paenibacillus_elgii            SPSNK-------------AELNGGGTGSGYTLHVTGDHWVIKDLKITNAK 121
Paenibacillus_alvei            DPSHP-------------AVLSGNSTSSGYAMRVRGDYWEIRDLKFTNAQ 126
SjAly_Saccharina_japonica      EGAKIKAGSPSVHIRHSWILLQGFTVDGKHESSSKKSSYVNKCVSVEGTG 145
                                  :                *.*  ... :      . :    :.. .:

                              128                      154 156
PsAly                          KGIMLDHANHVQMDGLEIYDIGDEGVHFRDGSSDNIIRNSHIYNTGLIEA 177
Paenibacillus_sp._YN15         KGIMLDHANNNWLEYLDVYNIGEEGVHFRDGSSNNVIKNSNVYDTGKLNP 164
Paenibacillus_ehimensis        KGIMLDHANYTLIDGAEVYQIGEEGVHYRDGSSYNTIRNSYVHDIGTVNP 171
Paenibacillus_elgii            KGIMLDHANYTLIDGAEVYQIGEEGVHYRDGSSYNTIRNSYLHDIGTVNP 171
Paenibacillus_alvei            KGIILDHSNYSLITDVEVYNTGMEGVHFRDGSSFSTIQNSSIHHTGRTAP 176
SjAly_Saccharina_japonica      K-SSSDPLKGFVMKDMVIKNCGTECVRLKNFVTNAEIKDNTITKCGVYDF 194
                               *    *  :   :    : : * * *: ::  :   *::. : . *

                                       181 184 188 191 195                216 219221
PsAly                          GF------GEGIYVGSDKGKWATYNKSADRNVISGVRIGPGVAAEHIDIK 221
Paenibacillus_sp._YN15         EY------GEGVYVGSDVGKWGSYKKETNNNRISAVSFGPNVRAEHIDIK 208
Paenibacillus_ehimensis        QY------GEAIYVGSDKGKWGTFNAATNYNTIANNTLGPNVAAEHIDIK 215
Paenibacillus_elgii            SF------GEAIYVGSDKGKWGTFNAATNHNTIANNTIGPNVAAEHIDIK 215
Paenibacillus_alvei            GY------GEGVYVGSADG--AGYNQATHHNTIRNVVFGPQVTAEHVDIK 218
SjAly_Saccharina_japonica      KFDEGGDNGEGIYIGTSSTQWKNGEDRCNDNRISGNTIS-TYGSECVDVK 243
                                :      **.:*:*:        :   . * *    :.    :* :*:*

                                                    244246 250 252   261
PsAly                          EGTVGTIVENSVFNGTGITGANYADSFIDVKGNDAVIRNNIGYRNGNSNI 271
Paenibacillus_sp._YN15         EGSSSTIVENCIFDGTGISGANYADSFIDVKGNNDIIRNNTGYRNGNSKI 258
Paenibacillus_ehimensis        EGSAGTLVENNTFDGTGMSGANAADSFIDVKGNNDVIRNNTGYRNGNSSI 265
Paenibacillus_elgii            EGSTGTLVENNTFDGTGMSGANAADSFIDVKGNNDVIRGNIGYRNGNSNI 265
Paenibacillus_alvei            ERTLGTIVENCIFNGEGISGANYADSFMDVKGNDAIIRNNVGYQEGNHTI 268
SjAly_Saccharina_japonica      EGCSGTTIEENKCSDQ----RQALTGCFSVRGDDNTVRYNT-AKDCEGVG 288
                               *   .* :*:   ..      :   . :.*:*::  :* *   :: :

                                 276 278           293                      318
PsAly                          VDAFQVHVQVAGWGQNATFTGNTVYLDQAAPYVVNAVGDATASAAGNQRY 321
Paenibacillus_sp._YN15         VDAFQVHERSSGWGQNAKFTGNTLYLDNATPYVVNADSGATATASNNTRS 308
Paenibacillus_ehimensis        KDAFQVHQRAAGWGQNASFTNNTVYLDNTTAYVVNAASGTTASASGNTRY 315
Paenibacillus_elgii            KDAFQVHQRAAGWGQNASFTNNTVYLDNTTAYVVNAASGTTASASSNTRY 315
Paenibacillus_alvei            IDAFQLHEVVTGWGSNNIFINNKVHMDNSEAYVIAAYNNANAKAANTIRT 318
SjAly_Saccharina_japonica      VRLGGAKVGKHQYGKDNDVYENTITDAEMGFMRITAMPQGRICENKCKGG 338
                                     :     :*.:  .  *.:   :     : *

PsAly                          PAGNLYQGHVNAAPAN--------- 337
Paenibacillus_sp._YN15         PSGNMYKGSVN-------------- 319
Paenibacillus_ehimensis        PAGNMYNGSVTSGEKTERPSASSSH 340
Paenibacillus_elgii            PAGNMYTGSVTGGK----------- 329
Paenibacillus_alvei            PSGNMYKGNITAYVTDVQLK----- 338
SjAly_Saccharina_japonica      SCDITADIGVRDIRETWDEDC---- 359
                               ...      :
```

**Figure S8. Amino acid sequence alignment of PsAly and other homologous proteins using the ClustalW program**

The four amino acid sequences of uncharacterised proteins of *Paenibacillus* were randomly selected from the 150 sequences used in the multiple amino acid sequence alignment by the ConSurf server. PsAly denotes the alginate lyase of *P*. str. FPU-7 (Accession no. LC490364). Paenibacillus_sp._YN15 denotes the protein of *Paenibacillus* sp. YN15 (Accession no. WP113020684, 61% identity between this protein and PsAly). Paenibacillus_ehimensis denotes the protein of *Paenibacillus ehimensis* (Accession no. WP025852769, 61% identity). Paenibacillus_elgii denotes the protein of *Paenibacillus elgii* (Accession no. WP063181803, 61% identity). Paenibacillus_alvei denotes the protein of *Paenibacillus alvei* (Accession no. WP081505786, 54% identity). SjAly denotes the alginate lyase of the brown alga *Saccharina japonica* (Accession no. BBH10631, 24% identity). The residues surrounded by red boxes (Glu181, Asp219, Glu216, Asp246, and Gln276) interact with cations on the cleft and are completely conserved among the proteins of *Paenibacilli*. The residues surrounded by yellow boxes (Lys128, His154, Arg156, Tyr184, Lys221, Asp250, and Lys252) are also completely conserved within the proteins of *Paenibacilli* on the cleft. The residues surrounded by cyan boxes (Asp188, Lys191, Tyr195, Tyr244, and His278) are highly conserved residues within the cleft. The residues surrounded by green boxes (Asn261, Asn293, and Asn318) form the Asn ladder in the β-helix fold.

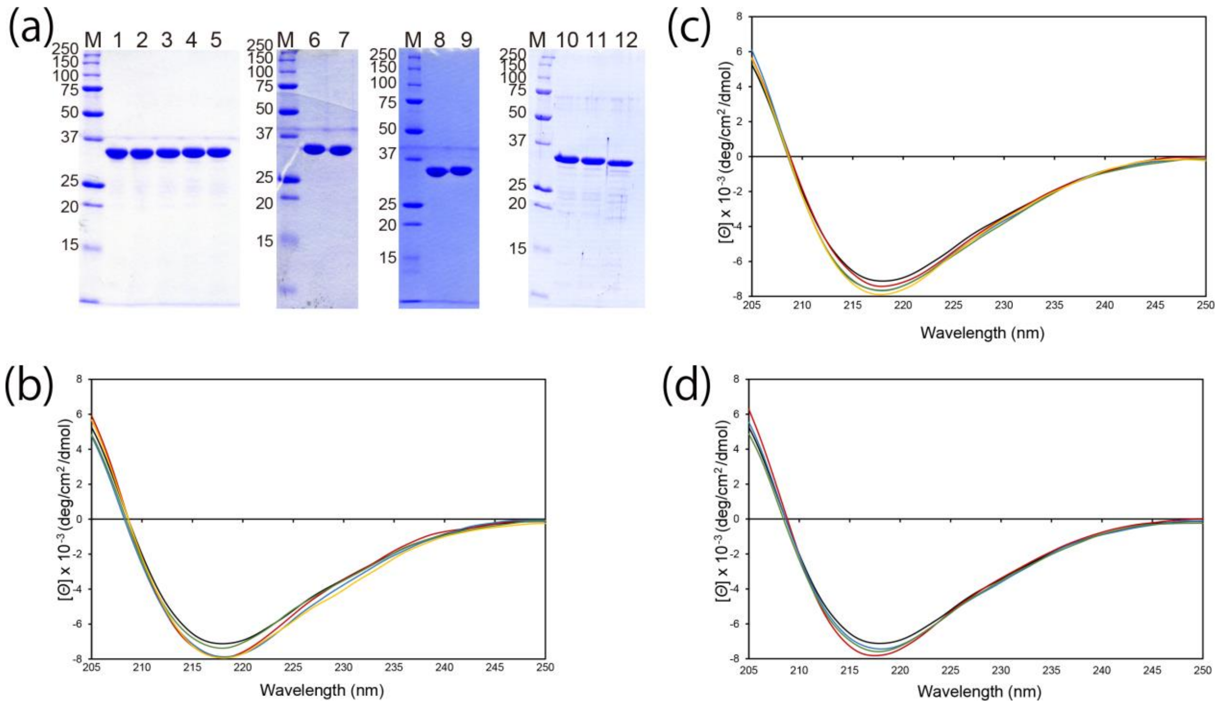


**Figure S9. SDS-PAGE profile (a) and far-UV circular dichroism (CD) spectra (b–d) of PsAly wild-type and mutant enzymes**

(a) Protein bands were stained with CBB R-250. Lane M, molecular mass standards; lane 1, purified PsAly; lane 1, purified PsAly wild-type (WT); lane 2, Y184; lane 3, D188N; lane 4, K221A; lane 5, K252A; lane 6, H278A; lane 7, Y244F; lane 8, K191A; lane 9, R156A; lane 10, Y195F; lane 11, K128A; lane 12, D250N. Each lane was loaded with 1 μg of enzyme. (b) The black curve indicates the far-UV CD spectrum of the WT; red, K128A; blue, R156A; green, Y184F; yellow, D188N. (c) Black, WT; red, K191A; blue, Y195F; green, K221A; yellow, Y244F. (d) Black, WT; red, D250N; blue, K252A; green, H278A.

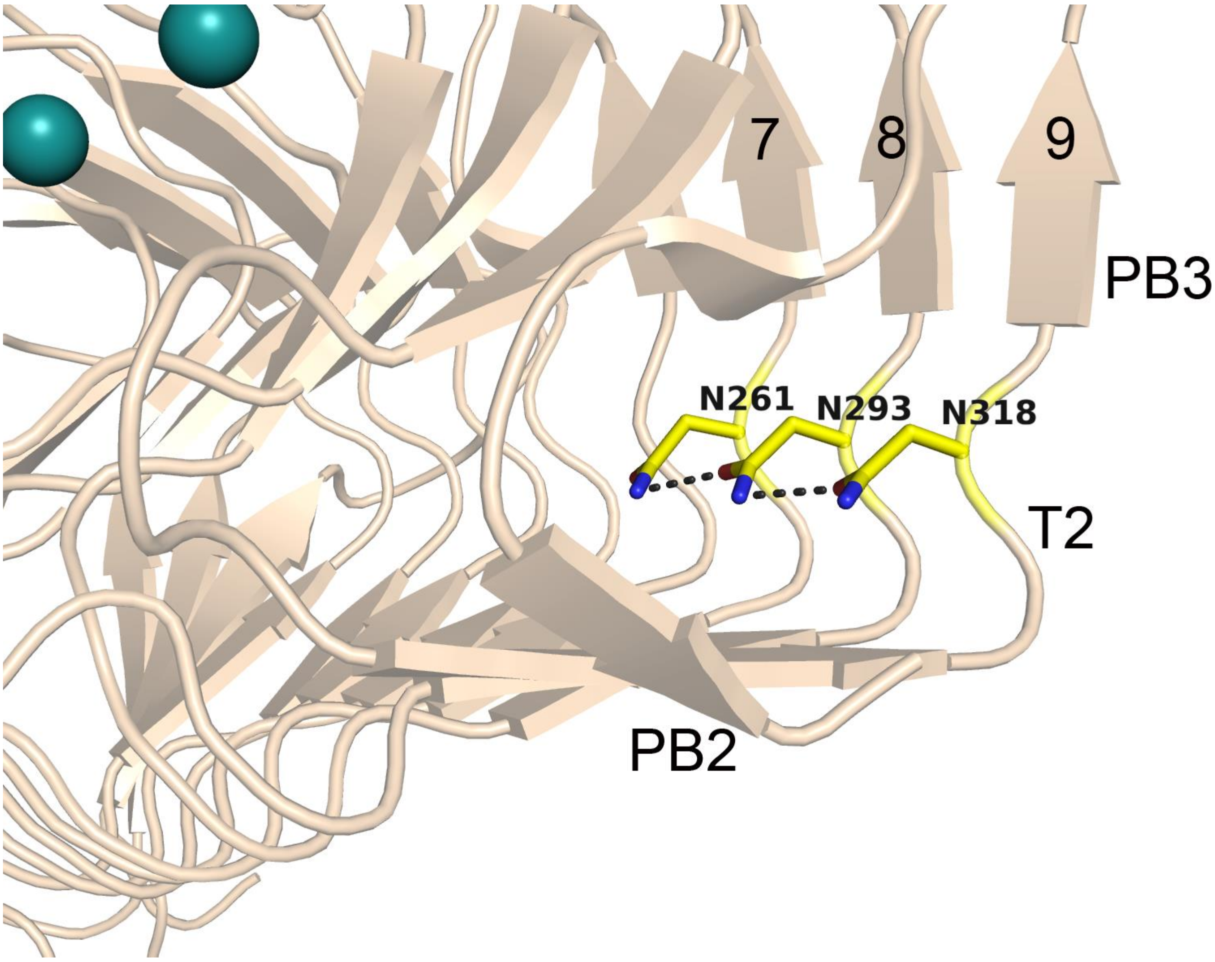


**Figure S10. The conserved amino acid residues in the inner structure of PsAly**

Three Asn residues, Asn261, Asn293, and Asn318, were stacked at T2 in the inner structure of PsAly as an Asn ladder commonly observed in the β-helix structure.

# Full wwPDB X-ray Structure Validation Report (i)

Sep 6, 2019 – 01:14 PM JST

| | | |
|---|---|---|
| PDB ID | : | 6KFN |
| Title | : | Crystal structure of alginate lyase from Paenibacillus sp. str. FPU-7 |
| Deposited on | : | 2019-07-08 |
| Resolution | : | 0.89 Å(reported) |

This is a Full wwPDB X-ray Structure Validation Report.

This report is produced by the wwPDB biocuration pipeline after annotation of the structure.

We welcome your comments at *validation@mail.wwpdb.org*
A user guide is available at
https://www.wwpdb.org/validation/2017/XrayValidationReportHelp
with specific help available everywhere you see the (i) symbol.

The following versions of software and data (see references (i)) were used in the production of this report:

| | | |
|---|---|---|
| MolProbity | : | 4.02b-467 |
| Mogul | : | 1.8.0 (224370), CSD as540be (2019) |
| Xtriage (Phenix) | : | 1.13 |
| EDS | : | 2.4 |
| buster-report | : | 1.1.7 (2018) |
| Percentile statistics | : | 20171227.v01 (using entries in the PDB archive December 27th 2017) |
| Refmac | : | 5.8.0158 |
| CCP4 | : | 7.0 (Gargrove) |
| Ideal geometry (proteins) | : | Engh & Huber (2001) |
| Ideal geometry (DNA, RNA) | : | Parkinson et al. (1996) |
| Validation Pipeline (wwPDB-VP) | : | 2.4 |

CONFIDENTIAL VALIDATION REPORT

# 1 Overall quality at a glance (i)

The following experimental techniques were used to determine the structure:
*X-RAY DIFFRACTION*

The reported resolution of this entry is 0.89 Å.

Percentile scores (ranging between 0-100) for global validation metrics of the entry are shown in the following graphic. The table shows the number of entries on which the scores are based.

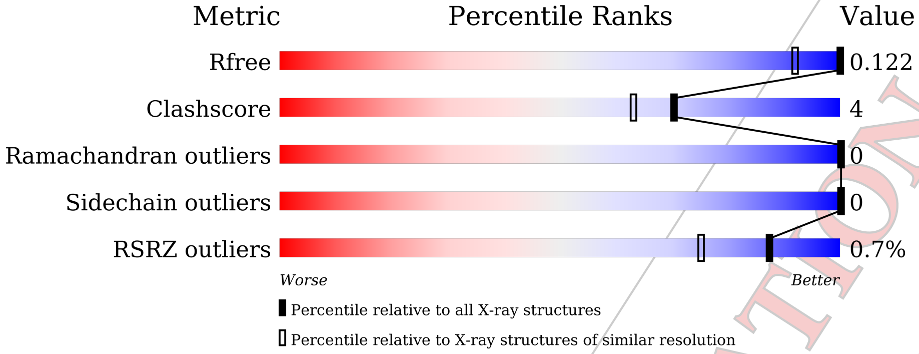


| Metric | Whole archive (#Entries) | Similar resolution (#Entries, resolution range(Å)) |
|---|---|---|
| $R_{free}$ | 111664 | 1087 (1.06-0.74) |
| Clashscore | 122126 | 1156 (1.06-0.74) |
| Ramachandran outliers | 120053 | 1069 (1.06-0.74) |
| Sidechain outliers | 120020 | 1071 (1.06-0.74) |
| RSRZ outliers | 108989 | 1054 (1.06-0.74) |

The table below summarises the geometric issues observed across the polymeric chains and their fit to the electron density. The red, orange, yellow and green segments on the lower bar indicate the fraction of residues that contain outliers for >=3, 2, 1 and 0 types of geometric quality criteria. A grey segment represents the fraction of residues that are not modelled. The numeric value for each fraction is indicated below the corresponding segment, with a dot representing fractions <=5% The upper red bar (where present) indicates the fraction of residues that have poor fit to the electron density. The numeric value is given above the bar.

| Mol | Chain | Length | Quality of chain |
|---|---|---|---|
| 1 | A | 307 | 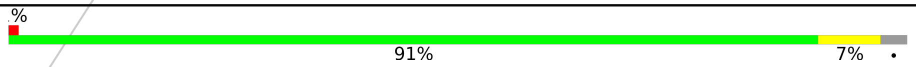 |

CONFIDENTIAL VALIDATION REPORT

## 2 Entry composition (i)

There are 4 unique types of molecules in this entry. The entry contains 4969 atoms, of which 2199 are hydrogens and 0 are deuteriums.

In the tables below, the ZeroOcc column contains the number of atoms modelled with zero occupancy, the AltConf column contains the number of residues with at least one atom in alternate conformation and the Trace column contains the number of residues modelled with at most 2 atoms.

- Molecule 1 is a protein called alginate lyase.

| Mol | Chain | Residues | Atoms | | | | | | ZeroOcc | AltConf | Trace |
|---|---|---|---|---|---|---|---|---|---|---|---|
| | | | Total | C | H | N | O | S | | | |
| 1 | A | 298 | 4522 | 1469 | 2195 | 399 | 453 | 6 | 0 | 26 | 0 |

- Molecule 2 is SODIUM ION (three-letter code: NA) (formula: Na) (labeled as "Ligand of Interest" by author).

| Mol | Chain | Residues | Atoms | | ZeroOcc | AltConf |
|---|---|---|---|---|---|---|
| | | | Total | Na | | |
| 2 | A | 2 | 2 | 2 | 0 | 0 |

- Molecule 3 is IMIDAZOLE (three-letter code: IMD) (formula: $C_3H_5N_2$).

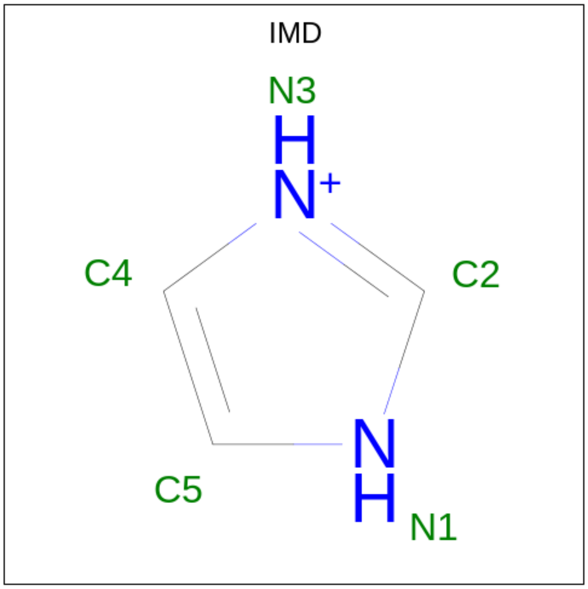


| Mol | Chain | Residues | Atoms | | | | ZeroOcc | AltConf |
|---|---|---|---|---|---|---|---|---|
| | | | Total | C | H | N | | |
| 3 | A | 1 | 9 | 3 | 4 | 2 | 0 | 0 |

- Molecule 4 is water.

CONFIDENTIAL VALIDATION REPORT

| Mol | Chain | Residues | Atoms | | ZeroOcc | AltConf |
|---|---|---|---|---|---|---|
| | | | Total | O | | |
| 4 | A | 433 | 436 | 436 | 0 | 3 |

CONFIDENTIAL VALIDATION REPORT

# 3 Residue-property plots (i)

These plots are drawn for all protein, RNA and DNA chains in the entry. The first graphic for a chain summarises the proportions of the various outlier classes displayed in the second graphic. The second graphic shows the sequence view annotated by issues in geometryand electron density. Residues are color-coded according to the number of geometric quality criteria for which they contain at least one outlier: green = 0, yellow = 1, orange = 2 and red = 3 or more. A red dot above a residue indicates a poor fit to the electron density (RSRZ > 2). Stretches of 2 or more consecutive residues without any outlier are shown as a green connector. Residues present in the sample, but not in the model, are shown in grey.

- Molecule 1: alginate lyase

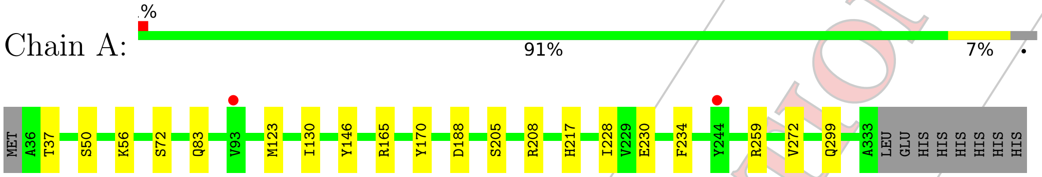


CONFIDENTIAL VALIDATION REPORT

# 4 Data and refinement statistics ⓘ

| Property | Value | Source |
|---|---|---|
| Space group | C 1 2 1 | Depositor |
| Cell constants<br>a, b, c, $\alpha$, $\beta$, $\gamma$ | 125.64Å 57.76Å 39.71Å<br>90.00° 91.10° 90.00° | Depositor |
| Resolution (Å) | 23.36 – 0.89<br>23.36 – 0.89 | Depositor<br>EDS |
| % Data completeness<br>(in resolution range) | 92.6 (23.36-0.89)<br>92.6 (23.36-0.89) | Depositor<br>EDS |
| $R_{merge}$ | 0.05 | Depositor |
| $R_{sym}$ | (Not available) | Depositor |
| $< I/\sigma(I) >$ [1] | 2.68 (at 0.89Å) | Xtriage |
| Refinement program | PHENIX 1.16_3549 | Depositor |
| R, $R_{free}$ | 0.105 , 0.122<br>0.106 , 0.122 | Depositor<br>DCC |
| $R_{free}$ test set | 9975 reflections (4.97%) | wwPDB-VP |
| Wilson B-factor (Å$^2$) | 6.3 | Xtriage |
| Anisotropy | 0.239 | Xtriage |
| Bulk solvent $k_{sol}$(e/Å$^3$), $B_{sol}$(Å$^2$) | 0.46 , 69.0 | EDS |
| L-test for twinning[2] | $< \|L\| > = 0.50$, $< L^2 > = 0.34$ | Xtriage |
| Estimated twinning fraction | 0.016 for -h,-k,l | Xtriage |
| $F_o$,$F_c$ correlation | 0.99 | EDS |
| Total number of atoms | 4969 | wwPDB-VP |
| Average B, all atoms (Å$^2$) | 12.0 | wwPDB-VP |

Xtriage's analysis on translational NCS is as follows: *The largest off-origin peak in the Patterson function is 7.14% of the height of the origin peak. No significant pseudotranslation is detected.*

[1] Intensities estimated from amplitudes.
[2] Theoretical values of $< |L| >$, $< L^2 >$ for acentric reflections are 0.5, 0.333 respectively for untwinned datasets, and 0.375, 0.2 for perfectly twinned datasets.

CONFIDENTIAL VALIDATION REPORT

# 5 Model quality (i)

## 5.1 Standard geometry (i)

Bond lengths and bond angles in the following residue types are not validated in this section: NA, IMD

The Z score for a bond length (or angle) is the number of standard deviations the observed value is removed from the expected value. A bond length (or angle) with $|Z| > 5$ is considered an outlier worth inspection. RMSZ is the root-mean-square of all Z scores of the bond lengths (or angles).

| Mol | Chain | Bond lengths | | Bond angles | |
|---|---|---|---|---|---|
| | | RMSZ | #$\|Z\|$ >5 | RMSZ | #$\|Z\|$ >5 |
| 1 | A | 0.60 | 0/2449 | 0.81 | 0/3322 |

There are no bond length outliers.

There are no bond angle outliers.

There are no chirality outliers.

There are no planarity outliers.

## 5.2 Too-close contacts (i)

In the following table, the Non-H and H(model) columns list the number of non-hydrogen atoms and hydrogen atoms in the chain respectively. The H(added) column lists the number of hydrogen atoms added and optimized by MolProbity. The Clashes column lists the number of clashes within the asymmetric unit, whereas Symm-Clashes lists symmetry related clashes.

| Mol | Chain | Non-H | H(model) | H(added) | Clashes | Symm-Clashes |
|---|---|---|---|---|---|---|
| 1 | A | 2327 | 2195 | 2340 | 17 | 0 |
| 2 | A | 2 | 0 | 0 | 0 | 0 |
| 3 | A | 5 | 4 | 5 | 0 | 0 |
| 4 | A | 436 | 0 | 0 | 12 | 4 |
| All | All | 2770 | 2199 | 2345 | 18 | 4 |

The all-atom clashscore is defined as the number of clashes found per 1000 atoms (including hydrogen atoms). The all-atom clashscore for this structure is 4.

All (18) close contacts within the same asymmetric unit are listed below, sorted by their clash magnitude.

CONFIDENTIAL VALIDATION REPORT

| Atom-1 | Atom-2 | Interatomic distance (Å) | Clash overlap (Å) |
|---|---|---|---|
| 1:A:50[B]:SER:OG | 4:A:501:HOH:O | 2.02 | 0.77 |
| 1:A:83[A]:GLN:OE1 | 4:A:502:HOH:O | 2.08 | 0.72 |
| 1:A:188[B]:ASP:OD1 | 4:A:503:HOH:O | 2.10 | 0.70 |
| 1:A:83[A]:GLN:CD | 4:A:502:HOH:O | 2.40 | 0.59 |
| 1:A:37:THR:OG1 | 1:A:83[B]:GLN:OE1 | 2.27 | 0.52 |
| 1:A:217[B]:HIS:ND1 | 1:A:234:PHE:HB3 | 2.25 | 0.51 |
| 1:A:299[A]:GLN:NE2 | 4:A:509:HOH:O | 2.30 | 0.49 |
| 1:A:56[B]:LYS:NZ | 4:A:514:HOH:O | 2.45 | 0.49 |
| 1:A:208[B]:ARG:HD3 | 4:A:547:HOH:O | 2.11 | 0.48 |
| 1:A:272[B]:VAL:HG11 | 4:A:909:HOH:O | 2.15 | 0.46 |
| 1:A:165:ARG:HA | 1:A:205:SER:O | 2.16 | 0.46 |
| 1:A:146:TYR:HA | 1:A:170:TYR:O | 2.17 | 0.45 |
| 1:A:123[B]:MET:SD | 1:A:130:ILE:HD11 | 2.57 | 0.44 |
| 1:A:228[B]:ILE:HD11 | 4:A:902:HOH:O | 2.17 | 0.44 |
| 1:A:83[B]:GLN:OE1 | 4:A:505:HOH:O | 2.20 | 0.43 |
| 1:A:72:SER:HA | 4:A:504:HOH:O | 2.19 | 0.42 |
| 1:A:230[A]:GLU:HA | 1:A:259:ARG:O | 2.20 | 0.40 |

All (4) symmetry-related close contacts are listed below. The label for Atom-2 includes the symmetry operator and encoded unit-cell translations to be applied.

| Atom-1 | Atom-2 | Interatomic distance (Å) | Clash overlap (Å) |
|---|---|---|---|
| 4:A:894:HOH:O | 4:A:894:HOH:O[2_556] | 1.66 | 0.54 |
| 4:A:684:HOH:O | 4:A:684:HOH:O[2_556] | 1.74 | 0.46 |
| 4:A:928:HOH:O | 4:A:928:HOH:O[2_555] | 1.90 | 0.30 |
| 4:A:547:HOH:O | 4:A:600:HOH:O[1_556] | 2.17 | 0.03 |

## 5.3 Torsion angles (i)

### 5.3.1 Protein backbone (i)

In the following table, the Percentiles column shows the percent Ramachandran outliers of the chain as a percentile score with respect to all X-ray entries followed by that with respect to entries of similar resolution.

The Analysed column shows the number of residues for which the backbone conformation was analysed, and the total number of residues.

| Mol | Chain | Analysed | Favoured | Allowed | Outliers | Percentiles |
|---|---|---|---|---|---|---|
| 1 | A | 323/307 (105%) | 316 (98%) | 7 (2%) | 0 | 100 100 |

CONFIDENTIAL VALIDATION REPORT

There are no Ramachandran outliers to report.

### 5.3.2 Protein sidechains (i)

In the following table, the Percentiles column shows the percent sidechain outliers of the chain as a percentile score with respect to all X-ray entries followed by that with respect to entries of similar resolution.

The Analysed column shows the number of residues for which the sidechain conformation was analysed, and the total number of residues.

| Mol | Chain | Analysed | Rotameric | Outliers | Percentiles | |
|---|---|---|---|---|---|---|
| 1 | A | 251/233 (108%) | 251 (100%) | 0 | 100 | 100 |

There are no protein residues with a non-rotameric sidechain to report.

Some sidechains can be flipped to improve hydrogen bonding and reduce clashes. There are no such sidechains identified.

### 5.3.3 RNA (i)

There are no RNA molecules in this entry.

## 5.4 Non-standard residues in protein, DNA, RNA chains (i)

There are no non-standard protein/DNA/RNA residues in this entry.

## 5.5 Carbohydrates (i)

There are no carbohydrates in this entry.

## 5.6 Ligand geometry (i)

Of 3 ligands modelled in this entry, 2 are monoatomic - leaving 1 for Mogul analysis.

In the following table, the Counts columns list the number of bonds (or angles) for which Mogul statistics could be retrieved, the number of bonds (or angles) that are observed in the model and the number of bonds (or angles) that are defined in the Chemical Component Dictionary. The Link column lists molecule types, if any, to which the group is linked. The Z score for a bond length (or angle) is the number of standard deviations the observed value is removed from the expected value. A bond length (or angle) with $|Z| > 2$ is considered an outlier worth inspection. RMSZ is the root-mean-square of all Z scores of the bond lengths (or angles).

CONFIDENTIAL VALIDATION REPORT

| Mol | Type | Chain | Res | Link | Bond lengths | | | Bond angles | | |
|---|---|---|---|---|---|---|---|---|---|---|
| | | | | | Counts | RMSZ | $\#\|Z\|>2$ | Counts | RMSZ | $\#\|Z\|>2$ |
| 3 | IMD | A | 403 | - | 3,5,5 | 0.76 | 0 | 4,5,5 | 0.46 | 0 |

In the following table, the Chirals column lists the number of chiral outliers, the number of chiral centers analysed, the number of these observed in the model and the number defined in the Chemical Component Dictionary. Similar counts are reported in the Torsion and Rings columns. '-' means no outliers of that kind were identified.

| Mol | Type | Chain | Res | Link | Chirals | Torsions | Rings |
|---|---|---|---|---|---|---|---|
| 3 | IMD | A | 403 | - | - | - | 0/1/1/1 |

There are no bond length outliers.

There are no bond angle outliers.

There are no chirality outliers.

There are no torsion outliers.

There are no ring outliers.

No monomer is involved in short contacts.

## 5.7 Other polymers (i)

There are no such residues in this entry.

## 5.8 Polymer linkage issues (i)

There are no chain breaks in this entry.

CONFIDENTIAL VALIDATION REPORT

# 6 Fit of model and data (i)

## 6.1 Protein, DNA and RNA chains (i)

In the following table, the column labelled '#RSRZ> 2' contains the number (and percentage) of RSRZ outliers, followed by percent RSRZ outliers for the chain as percentile scores relative to all X-ray entries and entries of similar resolution. The OWAB column contains the minimum, median, $95^{th}$ percentile and maximum values of the occupancy-weighted average B-factor per residue. The column labelled 'Q< 0.9' lists the number of (and percentage) of residues with an average occupancy less than 0.9.

| Mol | Chain | Analysed | <RSRZ> | #RSRZ>2 | | | OWAB(Å$^2$) | Q<0.9 |
|---|---|---|---|---|---|---|---|---|
| 1 | A | 298/307 (97%) | -0.76 | 2 (0%) | 87 | 75 | 4, 7, 20, 32 | 0 |

All (2) RSRZ outliers are listed below:

| Mol | Chain | Res | Type | RSRZ |
|---|---|---|---|---|
| 1 | A | 93 | VAL | 2.9 |
| 1 | A | 244 | TYR | 2.7 |

## 6.2 Non-standard residues in protein, DNA, RNA chains (i)

There are no non-standard protein/DNA/RNA residues in this entry.

## 6.3 Carbohydrates (i)

There are no carbohydrates in this entry.

## 6.4 Ligands (i)

In the following table, the Atoms column lists the number of modelled atoms in the group and the number defined in the chemical component dictionary. The B-factors column lists the minimum, median, $95^{th}$ percentile and maximum values of B factors of atoms in the group. The column labelled 'Q< 0.9' lists the number of atoms with occupancy less than 0.9.

| Mol | Type | Chain | Res | Atoms | RSCC | RSR | B-factors(Å$^2$) | Q<0.9 |
|---|---|---|---|---|---|---|---|---|
| 3 | IMD | A | 403 | 5/5 | 0.87 | 0.13 | 3,24,28,62 | 2 |
| 2 | NA | A | 402 | 1/1 | 1.00 | 0.02 | 5,5,5,5 | 0 |
| 2 | NA | A | 401 | 1/1 | 1.00 | 0.04 | 8,8,8,8 | 0 |

The following is a graphical depiction of the model fit to experimental electron density of all instances of the Ligand of Interest. In addition, ligands with molecular weight > 250 and outliers

CONFIDENTIAL VALIDATION REPORT

as shown on the geometry validation Tables will also be included. Each fit is shown from different orientation to approximate a three-dimensional view.

**Electron density around NA A 402:**

$2mF_o$-$DF_c$ (at 0.7 rmsd) in gray
$mF_o$-$DF_c$ (at 3 rmsd) in purple (negative) and green (positive)

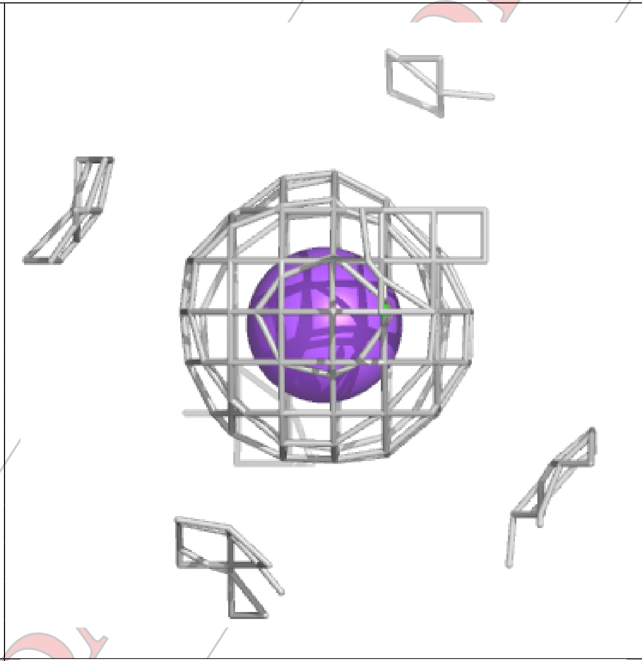

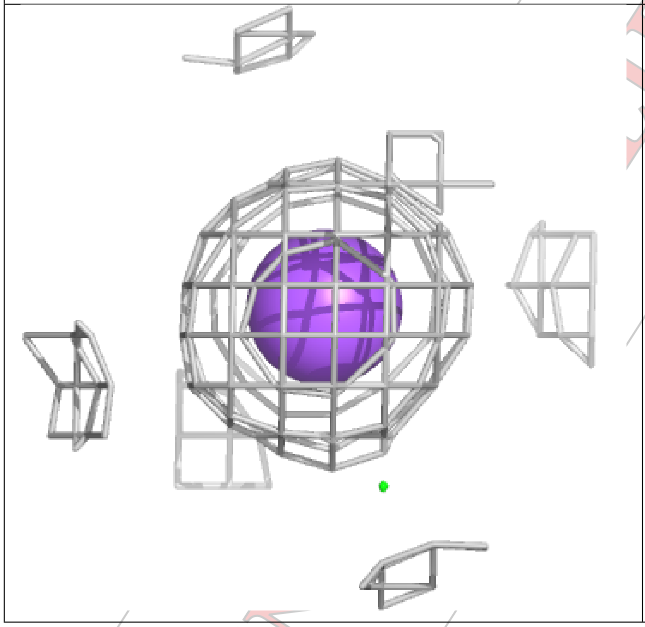

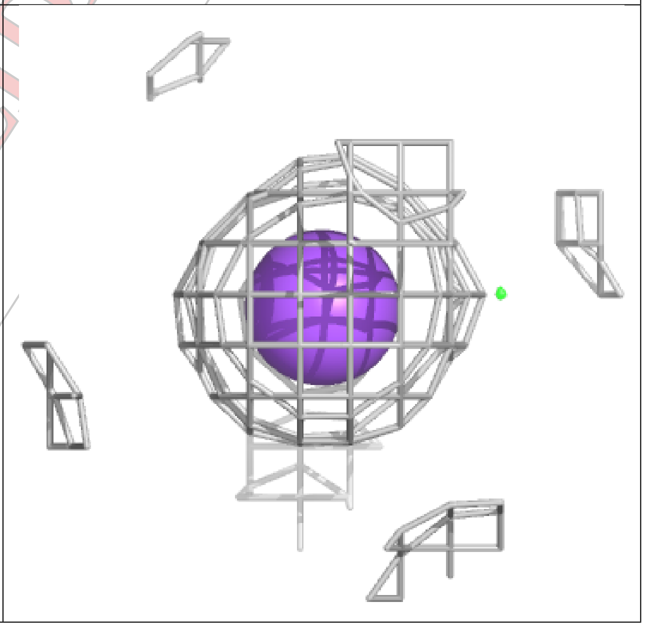

CONFIDENTIAL REPORT

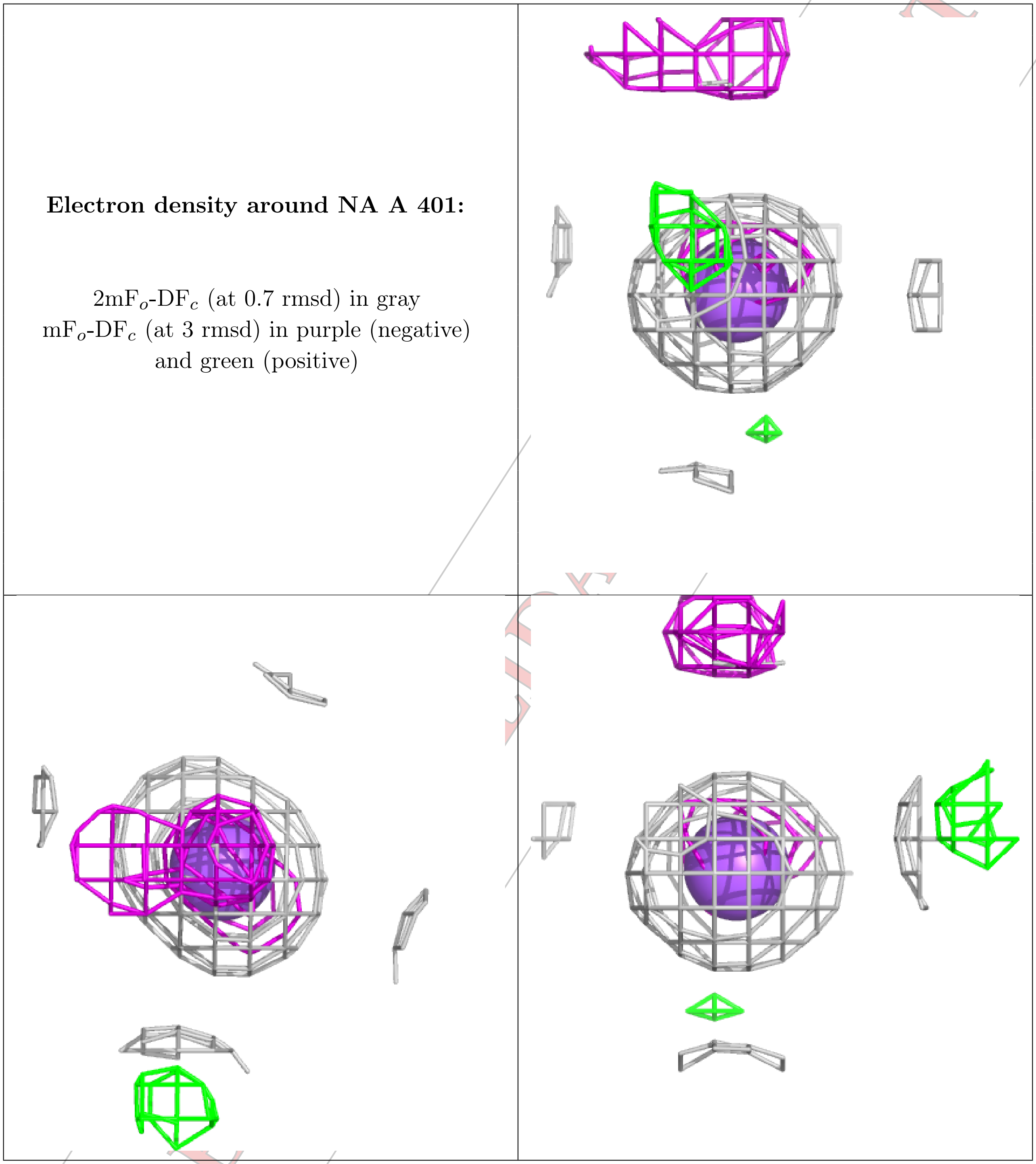

**Electron density around NA A 401:**

$2mF_o$-$DF_c$ (at 0.7 rmsd) in gray
$mF_o$-$DF_c$ (at 3 rmsd) in purple (negative)
and green (positive)

## 6.5 Other polymers ⓘ

There are no such residues in this entry.

CONFIDENTIAL
